# Ruminal microbiome-host crosstalk stimulates the development of the ruminal epithelium in a lamb model

**DOI:** 10.1186/s40168-019-0701-y

**Published:** 2019-06-03

**Authors:** Limei Lin, Fei Xie, Daming Sun, Junhua Liu, Weiyun Zhu, Shengyong Mao

**Affiliations:** 1 Laboratory of Gastrointestinal Microbiology, Jiangsu Key Laboratory of Gastrointestinal Nutrition and Animal Health, College of Animal Science and Technology, Nanjing Agricultural University, Nanjing, 210095 China; 2 National Center for International Research on Animal Gut Nutrition, Nanjing Agricultural University, Nanjing, 210095 China

**Keywords:** Starter feeding, Lamb, Rumen, Symbiotic microbiome, Crosstalk

## Abstract

**Background:**

The development of the rumen is an important physiological challenge for young ruminants. Previous studies have shown that starter feeding can effectively facilitate the growth and development of the rumen in ruminants. However, the mechanism through which starter feeding stimulates the development of the rumen is not clear. Here, we performed an integrated analysis in ruminal microbiota and a host transcriptomic profile in a lamb model with the intervention of starter feed to understand the ruminal microbiome-host crosstalk in stimulating the development of the ruminal epithelium.

**Results:**

Decreased ruminal pH and increased acetate and butyrate concentrations in the rumen, followed by increasing rumen organ index, were observed in lambs supplemented with starter. Using metagenome sequencing in combination with 16S rRNA and 18S rRNA gene amplicon sequencing, the results showed the abundance of acetate-producing *Mitsuokella* spp., lactate-producing *Sharpea* spp., lactate-utilizing *Megasphaera* spp., and *Entodinium* spp. was enriched in rumen microbial communities in the starter-feed group. The abundances of genes involved in sugar degradation were decreased in starter-feed lambs, but the GH13 encoding α-amylase was obviously increased. Rumen epithelial transcriptome analysis revealed that seven differentially expressed genes, including *MAPK1*, *PIK3CB*, *TNFSF10*, *ITGA6*, *SNAI2*, *SAV1*, and *DLG*, related to the cell growth module were upregulated, and *BAD*’s promotion of cell death was downregulated. Correlation analysis revealed that the increase in the concentrations of acetate and butyrate significantly correlated with the expression of these genes, which indicates acetate and butyrate likely acted as important drivers in the ruminal microbiome-host crosstalk.

**Conclusions:**

The present study comprehensively describes the symbiotic relationship between the rumen microbiota and the host in lambs after starter feeding. Our data demonstrates that the microbiome-driven generation of acetate and butyrate mediated the growth-related genes’ regulation of the growth-associated signalling pathway in the ruminal epithelium. These co-development networks regulated many physiological processes in the epithelium, including papillae morphology and rumen epithelial growth.

**Electronic supplementary material:**

The online version of this article (10.1186/s40168-019-0701-y) contains supplementary material, which is available to authorized users.

## Background

For ruminants, the rumen is important for the host’s metabolism, immunity, and health. Vast microbes colonize in the rumen including bacteria, archaea, fungi, and protozoa, which play important roles in diet fermentation and the host’s energy supply. The rumen is also a natural bioreactor, in which microbial extracellular enzymes catalyse the hydrolysis of refractory dietary plant fibre that is otherwise resistant to the animal’s endogenous digestive enzymes [1]. This unique microbial ecosystem leads to the development of mutualistic symbiosis between hosts and their microbial colonizers [2–4]. In this respect, the rumen is also a useful model of how ecosystems develop and operate because it is contained and yet susceptible to experimental manipulation, for instance by diverse dietary niches of the host organism [3, 5].

Most interestingly, the rumen epithelium is a unique place of interaction between host and microbial metabolism in that the rumen epithelium affects the net use of nutrients of the whole body, which physically serves as a barrier to the rumen epithelium’s contents, by providing VFA absorption capacity [6, 7]. Emerging evidence has suggested that the development of the ruminal epithelium is caused by the lifelong metabolic communication between the rumen microbiota and the host, which develops and changes with diet [6, 7]. Studies on the symbiotic relationship between ruminal microbiota and the development of the ruminal epithelium have shown that early intervention of starter feed, compared to intervention in adulthood, significantly stimulates the development of the rumen microbial community in neonatal ruminants and promotes the growth of ruminal papillae, which further benefit the absorption and metabolism of VFA in the ruminal epithelium [8–10]. This developmental process stimulated by starter feeding in neonatal ruminants is of great interest to researchers, as the process can be used as a model for the investigation of naturally occurring microbiota-host interactions. In investigations of the interactions between the ruminal microbiota and host, previous studies have mainly applied sequencing technologies to describe vast quantities of information related to microbial communication [9] or have used quantitative, real-time PCR or transcriptome sequencing to characterize a host’s expression of related genes [10].

To date, there have been few studies that explore the underlying mechanism of microbiome-host crosstalk in stimulating the development of ruminal papillae by diverse dietary niches. Here, to develop a deeper understanding of ruminal microbiome-host crosstalk in the development of the rumen epithelium, we used 16S rRNA gene, 18S rRNA gene, shotgun metagenome, and transcriptome sequencing techniques to explore the interactions between the ruminal microbiota and host. This study brings new insights into the interactions between the ruminal microbial population and the host and highlights the importance of the co-development of the host and its microbiota in terms of host fitness.

## Results

### Ruminal parameters concerning VFA profiles and papillae morphology

**Fig. 1:**
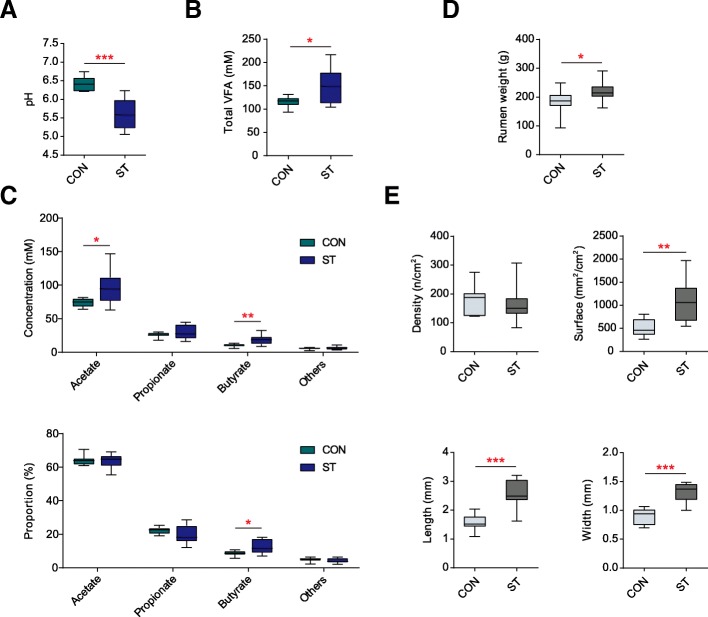
Effects of starter feeding on the rumen fermentation parameter: including lumen pH (**a**) and the concentration of total VFA (**b**). **c** Comparisons of the concentrations and proportion of acetate: propionate and butyrate in the lumen between the CON and ST groups (*n* = 10 per group). **d** Comparison of the rumen weight emptied the digesta between the two groups (*n* = 10 per group). **e** Comparisons of the parameters of rumen epithelial papillae between the two groups (*n* = 10 per group). *p < 0.05, **p < 0.01, ***p < 0.001

Compared with the control (CON) group (Fig. 1a–c; Additional file 2: Table S2), starter feeding decreased the ruminal pH (*p* < 0.001). The total VFA (*p* = 0.034), acetate (*p* = 0.028), and butyrate (*p* = 0.007) were increased after starter feeding, while propionate (*p* = 0.650) and other VFA (*p* = 0.496) remained unchanged. In the molar proportion, butyrate was higher in the group supplemented with starter feed (ST) (*p* = 0.019), whereas acetate (*p* = 1.000), propionate (*p* = 0.151), other VFA proportions (*p* = 0.326), and the acetate to propionate ratio (*p* = 0.131) were not significantly different between the two groups. As for the physiological indicators of the rumen, starter feeding increased the emptied weight of rumen (*p* = 0.034), as well as, increased the length (*p* < 0.001), width (*p* < 0.001), and surface (*p* = 0.001) of papillae in the ventral sac of the rumen (Fig. 1d, e; Additional file 3: Table S3) but did not affect the papillae density (*p* = 0.527).

### Taxonomic configurations of ruminal bacteria

**Fig. 2:**
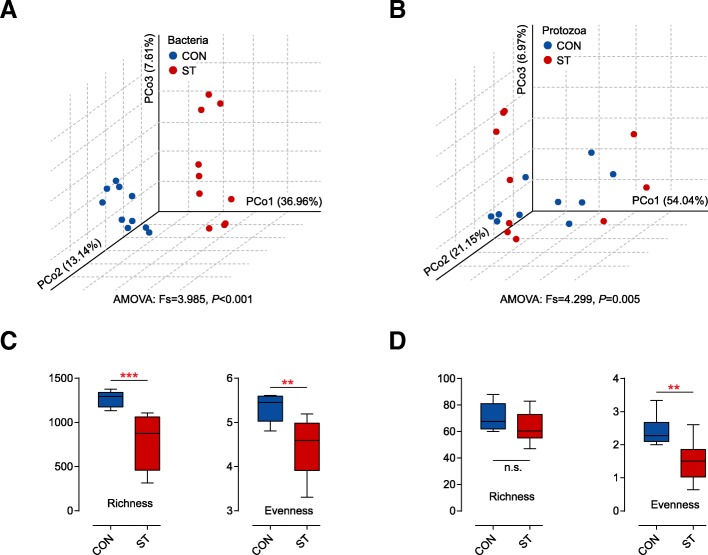
Principal coordinate analysis (PCoA) profile of ruminal bacterial diversity (**a**) and ciliate protozoal diversity (**b**) between the CON and ST groups (*n* = 10 per group) using a Bray-Curtis metric. AMOVA analysis showed significant differences between the two groups (*p* < 0.05). **c** Effects of starter feeding on the rumen bacterial richness (number of observed species) and evenness (Shannon diversity index values) at the 3% dissimilarity level. **d** Effects of starter feeding on the rumen ciliate protozoal richness (number of observed species) and evenness (Shannon diversity index values) at the 3% dissimilarity level. **p < 0.01, ***p < 0.001. n.s., not statistically significant

The bacterial structure profiles of the CON and ST groups were distinctly visualized by a PCoA plot (Fig. 2a). The Bray-Curtis metric revealed clear segregation and dissimilarities between the CON and ST groups based on 16S rRNA gene from 20 lambs (analysis of molecular variance (AMOVA); Fs = 3.985, *p* < 0.001). There was a total of 875,402 high-quality reads and an average of 43,770 ± 1737 reads per sample via 16S rRNA sequencing. Rarefaction curves approximately trended to a plateau at 27,942 reads revealed that the sequencing coverage was saturated (Additional file 4: Figure S1a). Compared with the CON group (Fig. 2c), starter feeding had significantly lower bacterial richness and evenness. All indices are shown in Additional file 5: Table S4.

**Fig. 3:**
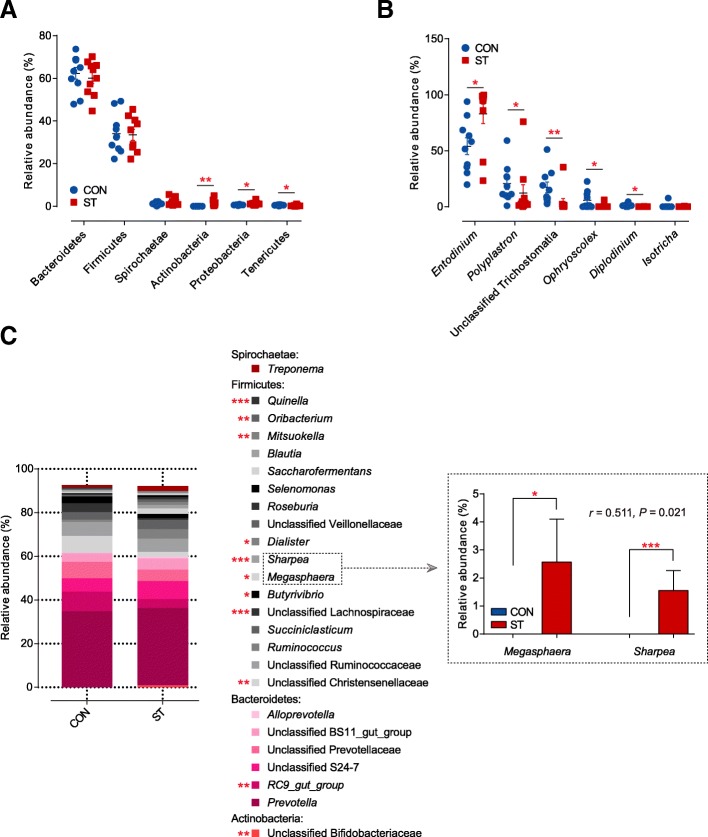
**a** Dominant phyla of bacteria that more than 0.5% at least one group were compared between the CON and ST groups (*n* = 10 per group). **b** Dominant genera of ciliate protozoa that more than 0.5% at least one group were compared between two groups. **c** Stacked bar graphs showing average percent reads of dominant genera of bacteria that more than 0.5% at least one group. The Spearman correlation coefficient and significance test based on the relative abundance of *Megasphaera* and *Sharpea* are shown on the right. *p < 0.05, **p < 0.01, ***p < 0.001

There were discriminatory features in the bacterial relative abundance at the phylum and genus levels with an identical threshold that mean relative abundance in one group was more than 0.5%. At the phylum level (Fig. 3a; Additional file 6: Table S5), most sequences were assigned into *Bacteroidetes* (62.35–60.15%) and *Firmicutes* (34.05–33.50%). Significant shifts were detected (*p* < 0.05) in three phyla, including *Proteobacteria*, *Tenericutes*, and *Actinobacteria*, during the period of starter feeding. Among these affected phyla, the relative abundance of *Proteobacteria* and *Actinobacteria* increased (*p* < 0.05), whereas the relative abundance of *Tenericutes* decreased (*p* < 0.05) in the ST group. At the genus level (Fig. 3c; Additional file 7: Table S6), the most predominant genus was *Prevotella* in the rumen. Of the 11 predominant taxa that significantly shifted during this study, the relative abundances of *Megasphaera*, *Sharpea*, *Dialiste*, *Mitsuokella*, and unclassified *Bifidobacteriaceae* were higher (*p* < 0.05) in the ST group. Moreover, the relationship between *Megasphaera* and *Sharpea* in terms of relative abundance was significantly strong (*r* = 0.511, *p* = 0.021). In addition, starter feeding significantly reduced the proportion of *RC9_gut_group*, unclassified *Christensenellaceae*, unclassified *Lachnospiraceae*, *Butyrivibrio*, *Oribacterium*, and *Quinella* compared to the CON group (*p* < 0.05; Additional file 7: Table S6).

**Fig. 4:**
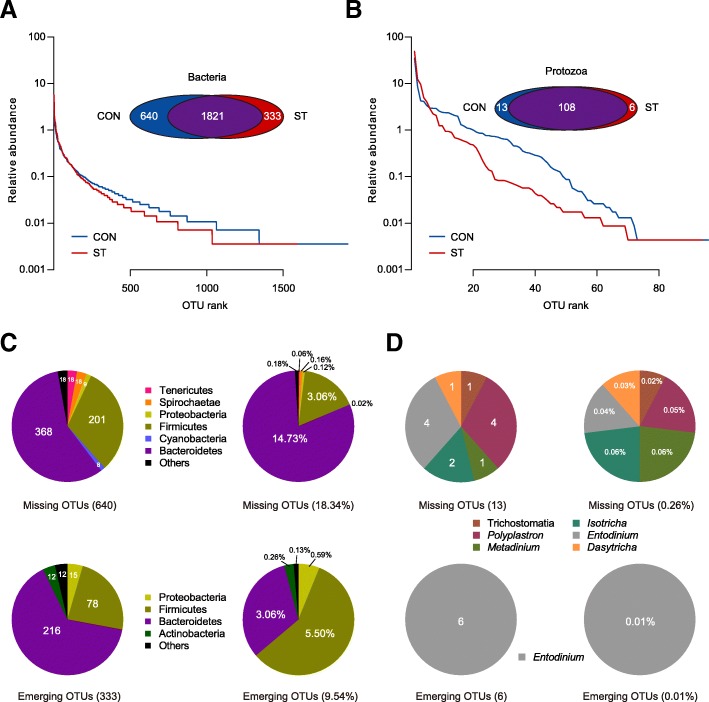
**a** Rank abundance curves and Venn diagram based on the average reads of bacteria community in the rumen of lambs (*n* = 10 per group). **b** Rank abundance curves and Venn diagram based on the average reads of the ciliate protozoa community in the rumen of lambs (*n* = 10 per group). **c** Pie charts showing the number and relative abundance of missing and emerging OTUs based on the OTU level of bacteria. The different colours of the parts represent the different taxonomic distribution of the OTUs at the phylum level. **d** Pie charts showing the number and relative abundance of missing and emerging OTUs based on the OTU level of the ciliate protozoa. The different colours of the parts represent the different taxonomic distribution of the OTUs at the family or genus level

Based on the above results, we identified all bacterial OTUs (Fig. 4a). In the OTU rank curve chart, the starter-feed lambs had a steeper curve, as above described by evenness. From the Venn profile, we can see that 1821 OTUs were shared between the two groups. In Fig. 4c, 368 missing OTUs being classified into *Bacteroidetes* that contributed to 14.73% in all OTUs, inversely emerging OTUs belonging to *Bacteroidetes* that only accounted for 3.06% compared with the CON group. Moreover, *Firmicutes* missed 201 OTUs contributing to 3.06% and emerged 78 OTUs accounting for 5.50%. Notably, the emerging OTUs, not the missing OTUs, were classified into *Actinobacteria*. This was in congruence with the shifted *Actinobacteria* (*p* = 0.002).

### Taxonomic configurations of ruminal protozoa

A PCoA plot (Fig. 2b) based on the Bray-Curtis metric revealed a segregation, and AMOVA analysis showed significant dissimilarities between CON and ST groups based on 18S rRNA gene from 20 lambs (Fs = 4.299, *p* = 0.005). After a quality filter was applied, 928,193 high quality reads and an average of 46,410 ± 2339 reads per sample were observed. Rarefaction curves approximately trended to a plateau at 22,952 reads, which revealed that the sequencing coverage was saturated (Additional file 4: Figure S1b). It was apparent that the diversity of ciliate protozoa between and within cohorts of co-located animals was much higher than the diversity in the ST cohort, as measured by species evenness (*p* = 0.001; Figure 2d; Additional file 8: Table S7), which is similar to our results from bacterial data, whereas no significant difference in species richness was observed (*p* = 0.069). Across the 18S rRNA gene samples, we found that almost all ciliate protozoal sequences were assigned to six genus-equivalent protozoal groups all belonging to *Ciliophora* with an identical threshold that means relative abundance in one group was more than 0.5% (Additional file 9: Table S8). In particular, we observed that the *Entodinium*, which dominated in the two cohort protozoa (54.05% in the CON group, 83.00% in the ST group), was an only significantly increased genus (*p* = 0.010). However, *Diplodinium*, *Ophryoscolex*, *Polyplastron*, and unclassified *Trichostomatia* were comparatively enriched in the CON group (*p* < 0.05; Fig. 3b). Then, screening out all ciliate protozoal OTUs (Fig. 4b), the starter-feed lambs had a steeper curve, as above described by evenness. Venn diagrams showed that 108 OTUs were shared between the two groups. In parallel, the CON group had more unique sequences (13 OTUs), followed by the ST (6 OTUs). Notably, all of the emerging OTUs belonged to the genus *Entodinium*, in agreement with its significantly changed abundance (Fig. 4d).

### Functions of the rumen microbiome

Based on the bacterial diversity, protozoal diversity, and Bray-Curtis metric, eight samples (Fig 2a, b) were selected and used for shotgun metagenome sequencing. After removing the reads mapped to the host, we obtained 158 Gb of paired-end sequencing data, which contained an average of 19.8 Gb (16.9–23.5 Gb) per sample. In total, a 3.8-Gb Pan-metagenome was constructed based on the assembled contigs with an average N50 length of 4.08 kb, including 4.3 million non-redundant genes, and the average length of ORF was 824 bp. Out of these unique genes derived from the rumen microflora, 70.8% genes were classified into eggNOG clusters, 3.2% genes were classified into CAZymes, 53.1% genes were identified as KO, and 44.9% genes were assigned to KEGG pathways.

### Carbohydrate genes related to starch degradation pathway

**Fig. 5:**
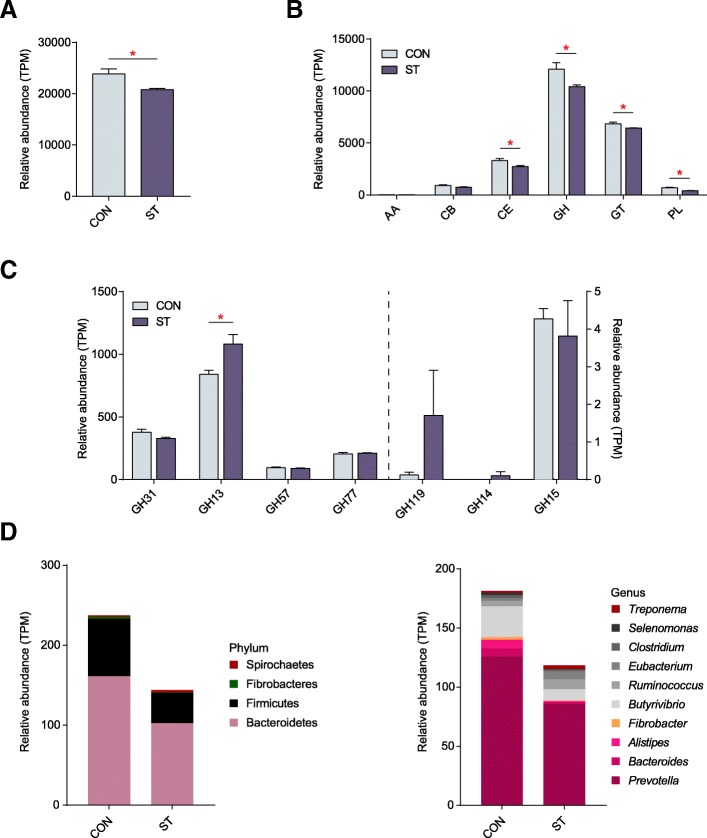
**a** Comparisons of the total abundance of CAZymes genes of rumen microbiomes of lambs in the CON and ST groups by the Mann−Whiney *U* test (*n* = 4 per group). **b** Comparisons of the relative abundance of the CAZymes gene families of the rumen microbiomes of lambs in the CON and ST groups (*n* = 4 per group). **c** Comparisons of the gene abundance of the GH family gene-coded amylolytic enzymes in the rumen of lambs in the CON and ST groups (*n* = 4 per group). *p < 0.05. **d** Phylogenetic distribution of sequences in GH13 assigned to the identified phylum and genus

The profiles of the CAZy families were strong predictors of diet in animals [11]. To specifically explore the microbial potential for dietary degradation in the CON and ST ruminal metagenomes, we screened for CAZymes in the assembled contigs. Here, a total of 136,424 unique genes obtained from metagenomics sequencing were searched against the CAZy database [12]. The ST group showed a lower abundance of CAZymes with respect to the CON group (*p* = 0.021, Mann−Whiney *U* test; Fig. 5a). Among those six classes of CAZymes families, there were significantly lower abundances of genes belonging to CEs, GHs, GTs, and PLs (Fig. 5b; Additional file 10: Table S9). These unique genes were assigned to 98 distinct families of GHs, 56 families of GTs, 14 families of PLs, 15 families of CEs, and 46 families of associated CBs, as well as 4 families of associated AAs. To further provide support for the pivotal starch biodegradation process, we screened for amylolytic enzymes, including α-amylase, β-amylase, and glucoamylase. In the sequence-based classification CAZymes of GH, we found α-amylases were grouped into the GH13, GH31, GH57, GH77, and GH119 families; β-amylase and glucoamylase were classified into the GH14 and GH15 families, respectively. Notably, among the gene encoding enzymes, the relative abundance of the GH13 family, as the largest sequence-based family of glycoside hydrolases, was significantly increased by starter feeding (*p* = 0.021); the others had no significant differences (Fig. 5c; Additional file 11: Table S10).

The higher abundance of genes coding GH13 in the ST groups prompted us to investigate these in more detail. Then, we determined the phylogenetic distributions based on the abundance of TPM assigned to each KO gene which were plotted at the phylum and genus levels. The top five carbohydrate-active enzymes classes (including GHs, GTs, PLs, CEs, and CBs) and the top 10 identified phyla or genera were linked based on the sequences (Additional file 12: Figure S2). Notably, the abundant GH13 genes with higher read counts in the ST group were mainly phylogenetically assigned to *Bacteroidetes* at the phylum level. In parallel, *Prevotella* and *Butyrivibrio* were the most assigned genera for the majority of genes enriched in the ST group (Fig. 5d).

### The fermentation pathways from glucose into acetate and butyrate by microorganisms

**Fig. 6:**
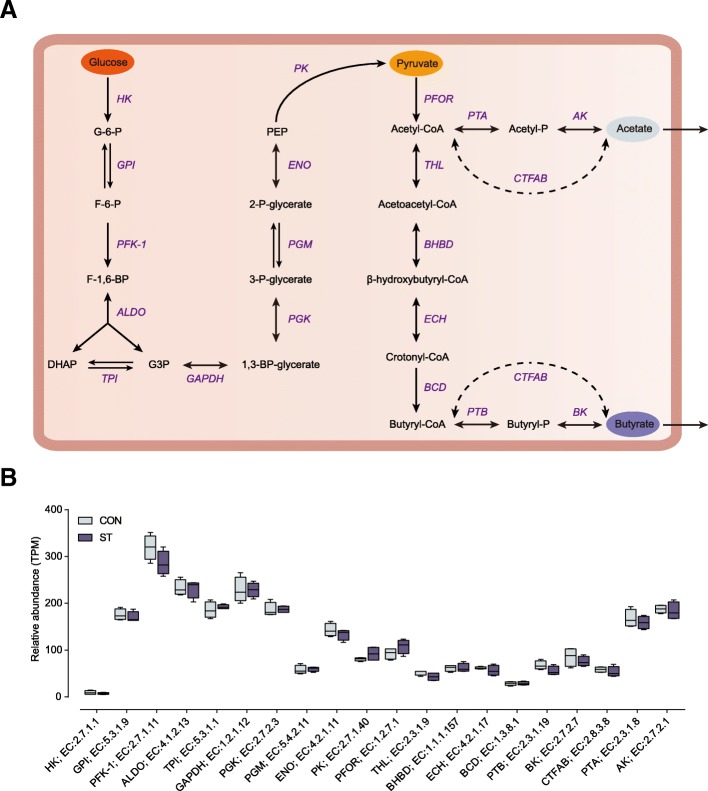
**a** Metabolic routes for butyrate and acetate production by direct conversion from carbohydrates. G, glucose; P, phosphate; F, fructose. **b** Comparisons of the relative abundance of KO enzymes related to the butyrate and acetate production pathway of lambs in the CON and ST groups by the Mann−Whiney *U* test (*n* = 4 per group). The lines inside the squares represent the median. There is no significant difference in all enzymes

Following the starch degradation pathway, many gene coding enzymes were involved in the acetate and butyrate fermentation pathways by microorganisms. We used metagenomic information to detect the potential function of the sequenced species on substrate utilization and fermentation. Then, the abundance of the KO genes related to the fermentation pathway was displayed between the CON and ST metagenomes. As shown in Fig. 6a, we screened for the fermentation pathway of metabolizing glucose into acetate and butyrate, which involved 21 encoding enzymes [13]. Interestingly, no significant differences in the abundance of the 21 enzymes were observed in the abundance based on metagenome sequencing between two groups (Fig. 6b).

### Transcriptome profiling analysis

**Fig. 7:**
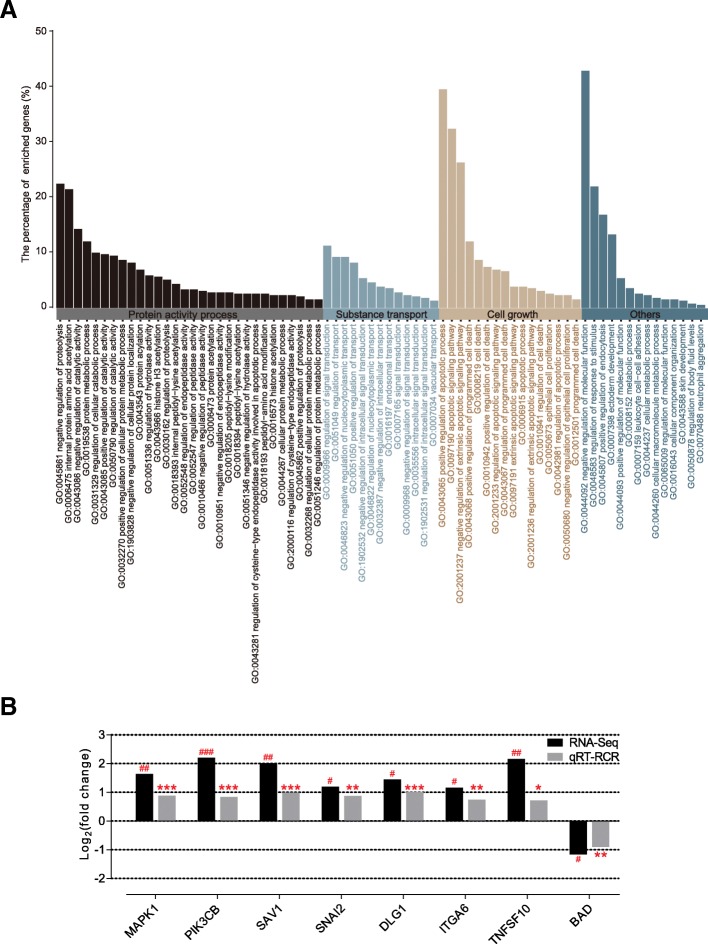
**a** Gene ontology analysis of genes based on DEGs. The enriched genes of 73 significantly altered GO terms include the regulation of three modules: protein activity processes (modification and degradation), substance transport, and cell growth (apoptosis and proliferation) are displayed on the histogram plot. Only terms with *p* < 0.05 were considered. **b** Differentially expressed genes related to the cell growth module in the rumen epithelium of lambs in the ST group (*n* = 4) compared with the CON group (*n* = 4). The values are presented as log_2_ (fold change). The *FDR* was calculated based on the *p* value. ^#^*FDR* < 0.05, ^##^*FDR* < 0.01, ^###^*FDR* < 0.001. qRT-PCR validation of transcriptomic results in the rumen epithelium of lambs in the ST group (*n* = 10) compared with the CON group (*n* = 10). The values are presented as log_2_ (fold change). *p < 0.05, **p < 0.01, ***p < 0.001

To investigate the differences in the host gene’s transcriptional level between the two groups, we performed transcriptome sequencing on total RNA samples from eight lambs (four lambs per group), whose ruminal DNA samples were used for the metagenomic analysis. We generated 54.82 Gb of clean data, with an average of 6.85 Gb (± 0.34 SEM) of clean data per subject. Among the encoded genes, 604 DEGs were identified from the comparison of the two groups (FDR < 0.05; fold change > 2). Among these DEGs, there were 358 upregulated genes and 246 downregulated genes. Using DAVID, the DEGs were then used to conduct GO enrichment analysis of the biological processes. We found that, among 73 significantly altered GO terms (*p* < 0.05), the regulations of three modules, namely protein activity processes (modification and degradation), substance transport, and cell growth (apoptosis and proliferation), were observed (Fig. 7a; Additional file 13: Table S11). As the development of the rumen epithelium was associated with the cell growth module, the KOBAS software was used to test the enrichment of DEGs in different KEGG pathways. The results showed that eight genes enriched in growth regulation modules were involved in the growth-associated signalling pathway. We found that *MAPK1*, *PIK3CB*, *SAV1*, *SNAI2*, *DLG1*, *ITGA6*, and *TNFSF10* were significantly upregulated in the ST group; inversely, starter feeding significantly downregulated *BAD* (Additional file 14: Table S12). The expression of these eight host genes was validated using quantitative PCR, and the expression trends remained consistent (Fig. 7b).

### Correlation between the microbial metabolites and rumen epithelium growth

**Fig. 8:**
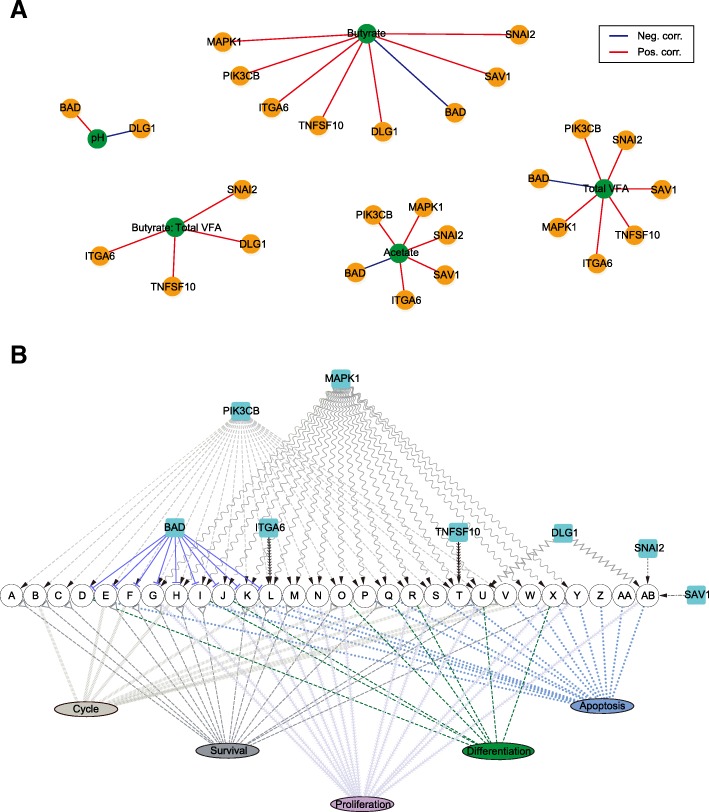
**a** The Spearman correlation coefficient revealed the association between the changes of fermentation parameters and the expression of these eight growth-related genes (SCC > 0.85 and *p* < 0.01). The lines’ colour represents two kinds of correlation: blue, negative correction and red, positive correction. **b** The co-regulation network of these eight genes is related to rumen epithelium growth. The arrow represents the activation of the signalling pathway, and the horizontal line represents the inhibition of the signalling pathway. The signalling pathways and functions involved in the network were predicted by the KEGG pathway analysis. The different colour lines represent different functions that these pathways regulated. Abbreviations: A, B cell receptor signalling pathway; B, Jak-STAT signalling pathway; C, mTOR signalling pathway; D, cGMP-PKG signalling pathway; E, ErbB signalling pathway; F, Ras signalling pathway; G, VEGF signalling pathway; H, thyroid hormone signalling pathway; I, neurotrophin signalling pathway; J, insulin signalling pathway; K, cAMP signalling pathway; L, PI3K-Akt signalling pathway; M, Rap1 signalling pathway; N, prolactin signalling pathway; O, phospholipase D signalling pathway; P, oestrogen signalling pathway; Q, chemokine signalling pathway; R, oxytocin signalling pathway; S, HIF-1 signalling pathway; T, FoxO signalling pathway; U, T cell receptor signalling pathway; V, TNF signalling pathway; W, TGF-beta signalling pathway; X, MAPK signalling pathway; Y, sphingolipid signalling pathway; Z, NF-kappa B signalling pathway; AA, RIG-I-like receptor signalling pathway; AB, hippo signalling pathway

To explore the potential microbiota-host metabolic communication, Spearman’s rank correlations were constructed between the fermentation parameters and the expression of the growth-related genes involved in the signalling pathway in the ruminal epithelium. The results revealed strong correlations with a threshold of SCC > 0.85 and *p* < 0.01. Individual VFA and rumen epithelial growth-related genes involved in the signalling pathway were identified. As shown in Fig. 8a, VFA greatly affected the expression of growth-related genes involved in the growth-associated signalling pathway. pH positively correlated with *BAD* and negatively correlated with *DLG1*. Besides, butyrate, acetate, and total VFA all had positive correlations with *MAPK1*, *PIK3CB*, *ITGA6*, *SNAI2*, and *SAV1*, whereas the expression of *BAD* negatively correlated with the expression of butyrate, acetate, and total VFA. There also existed other significant correlations, such as the positive correlations between butyrate and *TNF10*/*DLG1* and between total VFA and *TNF10*. Likewise, the proportion of butyrate significantly influenced many genes, including *TNFSF10*, *ITGA6*, *SNAI2*, and *DLG*. In short, these relationships indicated that VFA acts as a microbial metabolite to regulate microorganism-host systematic co-development on growth.

Our study identified a strong metabolic interaction between concentrations of individual VFA and the expression of the growth-related genes involved in the growth-associated signalling pathway in the ruminal epithelium. To search the axes of the host channels affected by microbial metabolites in more detail, the growth-associated signalling pathways in which the eight growth-related genes were involved in were screened; then, the gene-pathway-function co-expression network was described (Fig. 8b). The upregulated expressions of *PIK3CB* and *MAPK1* regulate cell proliferation, apoptosis, differentiation, cycle, and survival via 22 signalling pathways and 18 signalling pathways, respectively. *DLG1*, which was upregulated, was in the T cell receptor signalling pathway and the Hippo signalling pathway, which regulate cell proliferation, cell apoptosis, and cell differentiation. Moreover, the increased expressions of *TNFSF10*, *ITGA6*, *SNAI2*, and *SAV1* were all in the only signalling pathway in the regulation of cell growth. The downregulated genes, namely *BAD*, were located in nine signalling pathways for adjusting cell processes.

## Discussion

Currently, there is limited knowledge of the crosstalk between the rumen microbiome and host in stimulating the development of the ruminal epithelium when facing diverse dietary niches. In this study, we integrated rumen taxonomic configurations, metagenome analysis, and epithelial transcriptome profiling to explore the interactions between the ruminal microbiota and host. Previous studies on neonatal lambs [14, 15] have revealed that VFA greatly promotes rapid ruminal papillae development. Corresponding to this, the present study also showed that starter feeding significantly increased the concentrations of total VFA [16], which contained increasing concentrations of acetate and butyrate [17] and stimulated the development of the ruminal epithelium. Previously, this phenomenon of increased total VFA was congruent with the increase of starch researched on neonatal ruminants [18], which lacked accurate and mechanical elucidation for the reasons of increased VFA.

In addition, our data also revealed that the increased concentrations of acetate and butyrate were associated with changes in the rumen taxonomic fingerprints. Phylogenetic analysis of detectable microbial genera exhibited the increased abundance of five genera, namely *Mitsuokella*, *Sharpea*, *Megasphaera*, *Dialiste*, and unclassified *Bifidobacteriaceae*. Among them, intriguingly, *Mitsuokella* can ferment a wide range of carbohydrates and its major metabolic product was acetate [19–21]. *Sharpea* is known to be the lactate-producer, and *Megasphaera* can converse the lactate to butyrate [13, 22]. Additionally, there was a strong relationship between these two genera. Thus, *Sharpea* was accompanied by a corresponding increase in the percentage of *Megasphaera*, which increased butyrate production. Besides, unclassified *Bifidobacteriaceae*, as rumen starch-degradation bacteria, can promote the production of acetate and lactate [23]. Taken together, these findings were strongly consistent with significantly increased the level of acetate and butyrate, which indicates that the specific functional taxonomic groups stabilized the microbial ecosystem by preventing the accumulation of lactate and tend to produce more acetate and butyrate, thus promoting rumen epithelial growth. As for protozoa taxonomic fingerprints, phylogenetic, an important corollary, is that the most abundant presence of *Entodinium* significantly increased to 83% in the starter-feed group. A previous study showed that *Entodinium* engulfed starch granules, converted the digestion products to reserve carbohydrates and then maintained ruminal integrity fermentation [24–26]; this process was mirrored by the high amount of starch in our starter feed. Thus, the swallowing of starch granules by increased *Entodinium* limits bacterial rapid fermentation of starch and then stabilizes the pH oscillation to maintain rumen homeostasis, which is also beneficial for the conversion of lactate to butyrate [22].

The overwhelming data of starter feeding on the microbiota functional profiling have been reported based on PICRUSt [9]. However, the original implementation of PICRUSt is primarily from wide selection of microbial genomes in affiliated with the human microbiome, which may reduce the accuracy of functional predictions when applied to data from ruminant microbiomes. In our study, we used shotgun metagenome sequencing to perform the entire metabolic process from the starch degradation pathway to the acetate and butyrate fermentation pathways produced by microorganisms. Regarding the CAZymes, dramatically, family GH13 at high abundance, which is known as an α-amylase family that binds and degrades starch [11], occurred predominantly in genus *Prevotella*, and *Butyrivibrio* had a significantly higher abundance of genes. Consistent with this, the presence of *Prevotella* and *Butyrivibrio* in the starch-enriched diet of animals indicated that these genera may be related to the breakdown of complex polysaccharides [27, 28]. The finding also demonstrated the enrichment of GH13, which co-varied along with the increasing abundance of protozoa related to *Entodinium*, which plays a starch-biodegradation role in the rumen after starter feeding. This observation may be supported by the fact that several taxa and enzymes can converge to serve necessary functions for diverse dietary niches [29]. Thus, the increased enzyme and taxa facilitated the degradation from starch to glucose, which in turn facilitated the bioproduction of acetate and butyrate by the microorganisms. As for ruminal microbial fermentation pathways, these pathways are mainly mediated by microorganisms, which can ferment glucose to yield VFA that is absorbed by the rumen epithelium and promotes the ruminant fitness [13, 30]. Studying the fermentation process from glucose to acetate and butyrate under the catalysis of various enzymes found no significant differences in the abundance of the total 21 enzymes that were observed at the metagenome level between the two groups. Therefore, the increased starch content and the enrichment of GH13 with a starch-degrading capacity, not the shift in the catalysis of various enzymes involved in the fermentation pathway, resulted in promoting acetate and butyrate production in the ST group. Altogether, these findings indicate that starter feeding altered the composition and function of ruminal microbiota, which stabilized the microbial ecosystem and produced more acetate and butyrate.

Previous research on neonatal lambs [14, 15] revealed that VFA, especially butyrate, can rapidly promote ruminal papillae development; however, there is a paucity of data concerning the mechanisms through which the VFA mediates regulation in the growth of the rumen epithelium. GO enrichment analysis of the biological processes revealed that there are 73 significantly altered GO terms. This mainly included protein activity processes, substance transport, and cell growth. Among these three parts, protein activity processes mainly include histone acetylation and proteolysis. Burgeoning evidence indicates that butyrate mediates the decrease of histone acetylation centred on the transcription start site and the downregulation of associated genes [31], and proteolysis can regulate a diverse array of ion channels [32]. Thus, proteolysis was cross-talk with substance transport function and likely related to the transport and absorption of VFA. Of note, the chief biological processes were functionally annotated as being involved in cell growth, and this was confirmed by the increased rumen weight and rumen papillary development as outlined above. As already reported, early intervention of starter feed significantly promotes rumen epithelium development. Thus, of particular interest, we focus on the expression profile of eight growth-related genes involved in the signalling pathway in rumen epithelium. Among them, we found that *MAPK1*, *PIK3CB*, *TNFSF10*, *ITGA6*, *SNAI2*, *SAV1*, and *DLG1* were significantly upregulated; inversely, starter feeding significantly downregulated the expression of *BAD*. Additional support for this finding includes, for example, *MAPK1* [33] and *PIK3CB* [34], which are involved in multiple signalling pathways associated with cell growth. *SAV1* was reported to be an upstream Hippo signalling pathway regulator *in vivo* by functioning as a dual regulator of cell proliferation and apoptosis [35, 36], and increased *SAV1* may motivate the function of a downstream binding target, *SNAI2*, which lies in the Hippo signalling pathway of pro-proliferation and anti-apoptosis [37], thus promoting cell growth to stimulate the development of the rumen epithelium. In addition, *DLG1* shares homology with the *KIAA0008*, a membrane-associated GK domain protein that is vital for cellular growth acting as a cell cycle-regulated gene, thus the upregulated *DLG1* may have accelerated cell cycle progression [38]. *ITGA6* is involved in mediating the signalling pathways concerning cell adhesion and cell surface, including cell proliferation, migration, and invasion, and inhibits cell apoptosis [39]. Additionally, *BAD* and *TNFSF10* are both involved in the apoptotic process. *BAD* is a heterodimeric partner of *Bcl-XL* and *Bcl-2*, which can displace *Bax* and promote cell death [40]; thus, downregulated *BAD* may inhibit apoptosis to hasten physiological growth. *TNFSF10* can induce apoptotic signalling pathways, which control cell death, accelerate cell renewal, and advance physiological processes via upregulated gene expression [41]. Taken together, the starter feeding altered the expression of the growth-related genes, which regulated growth-associated with the signalling pathway in the ruminal epithelium and then stimulated the development of the rumen epithelium. In order to elucidate the relationship between the VFA and these genes outlined above, correlation analysis was carried out, and the results revealed highly positive correlations between acetate/butyrate and upregulated growth-related genes, whereas there was a highly negative correlation between acetate/butyrate and *BAD*. These results, combined with a previous report [14, 15], further demonstrated that the microbiome-driven generation of acetate and butyrate mediated the growth-related genes’ regulation of various growth-associated signalling pathways to promote rumen epithelium development. These findings also suggest that deliberately modulating microbiota-host metabolic and signal interactions to stimulate ruminal development, such as starter feeding, is fundamental to animal physiology.

## Conclusion

**Fig. 9:**
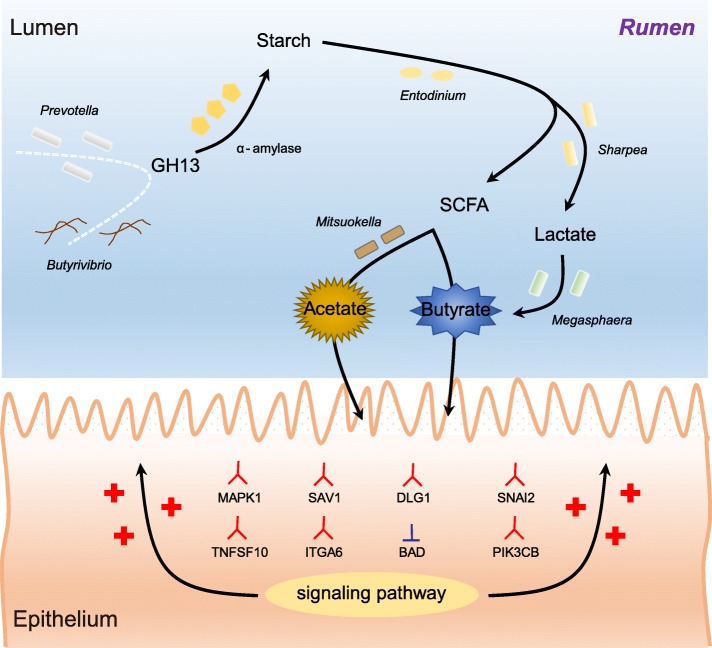
Proposed model of the generation of VFAs and the mediation for the growth-related genes in the rumen epithelium of lambs after the starter feeding. The red branch represents activation, and the inverted-T represents inhibition. The plus sign represents the growth of the rumen epithelium

Our results demonstrate the abundance of bacterial taxa with acetate-butyrate producing capacity and protozoal taxa with starch-degradation ability was increased by starter feeding in rumen taxonomic configurations. For microbial metabolic routes, starter feeding increased the abundance of genes that coded α-amylases, illustrating the increased production of acetate, and butyrate production was definitely related to the starch degradation pathway. The microbiome-driven generation of acetate and butyrate had a strong correlation with the growth-related genes locating in growth-associated signalling pathways in the ruminal epithelium. These co-development networks regulate multiple physiological processes in the ruminal epithelium, specifically rumen papillae morphology (Fig. 9). This co-development of host and its microbiota also provides a series of windows for dietary intervention in early life.

## Methods

### Experimental design

This study was conducted in accordance with the guidance of the Animal Care and Use Committee of Nanjing Agricultural University (SYXK (Su) 2015-0656) in China. We used 20 healthy 10-day-old *Hu* sheep in this experiment, which was performed at a stud farm in Jiangsu Province from October to December. The control group (CON) consisted of 10 lambs randomly kept with the dam, and the lambs were fed breast milk without receiving the starter feed. The starter treatment group (ST) consisted of the other 10 lambs that were fed breast milk for 1 h at 0630, 1030 and 1530 h, and also daily fed the starter feed ad libitum from 0400 to 1900 h in individual pens. The amount of DMI of the starter feed was targeted to 200 g·animal^−1^·d^−1^ throughout the experimental period, and the amounts of feed offered and residue were recorded daily for calculation of the DMI. To meet the nutrient requirement of *Hu* sheep lambs, we designed the formula of starter feed (Ministry of Agriculture of China, 2004). The ingredients and nutrient composition of the starter feed is provided in Additional file 1: Table S1. Oat hay (10.05% CP, 28.71% CF) and fresh water were given freely to all lambs—from 10-day-old lambs to 56-day-old lambs. None of the lambs had an opportunity to obtain the ewes’ feed. Every week, live body weight of all lambs before the morning feeding was measured. The experiment lasted 46 days, and all the lambs were slaughtered when they were 56-day-old.

### Sample collection

On the last day of the experimental period, all lambs were slaughtered 3 h after the morning feeding. The lambs were stunned and exsanguination immediately followed. The internal organs were immediately dissected and the rumen was separated; the content within the rumen was collected for sampling. Five milliliters of homogenized ruminal content samples were collected in triplicates and stored at − 20 °C for microbial DNA extraction. The other aliquot of the ruminal content sample was screened through a four-layer cheesecloth. Immediately, we used a portable pH meter (HI 9024C; HANNA Instruments, Woonsocket, RI) to measure the pH of the rumen fluid. Then, the fluid was kept in 25% (*w*/*v*) metaphosphoric acid (5 mL ruminal fluid: 1 mL metaphosphoric acid) and mixed and stored at − 20 °C for analysis of VFA concentrations in gas chromatography (GC-14B, Shimadzu, Japan) [42]. Subsequently, three segments of the epithelial tissue from the ventral sac of the rumen were excised from the muscular and serosal layers by blunt dissection, and then immediately washed in ice-cold PBS until the PBS was clear. One ruminal epithelial sample was fixed in 4% PFA (Sigma, USA) for histomorphometric microscopy analysis. Another ruminal epithelial sample (5 × 5 cm) was immediately fixed in cold PBS to measure papillae density and size. The last epithelial sample after detachment from the muscular and serosal layers by blunt dissection was cut to approximately 0.5 × 0.5 cm and then directly transferred into liquid nitrogen until tissue RNA extraction.

### DNA extraction and sequencing

The DNA of the rumen microbiota was extracted from 0.3 g ruminal content per sample using a DNA Kit (E.Z.N.A.® Soil DNA Kit, Omega Biotek, USA). The specific operation was strictly in accordance with the standard process specification of the DNA Kit. A bead-beating procedure was used to break down the cell wall of the microbes and separate the DNA. The purity and concentration of the obtained DNA was determined by a NanoDrop 1000 spectrophotometer (Nyxor Biotech, Paris, France). All the DNA samples were stored at − 80 °C until subsequent processing.

Through sequencing the region of bacterial 16S rRNA gene and ciliate protozoal 18S rRNA gene, we obtained the structure of the rumen microbial communities. For bacteria, we used universal primers to amplify the V3 and V4 regions of 16S rRNA gene and with a 6 bp barcode unique to each sample. The primers were (341F: 5′-CCTAYGGGRBGCASCAG-3′, 806R: 5′-GGACTACNNGGGTATCTAAT-3′). For protozoa, we used special primers to amplify the ciliate protozoal 18S rRNA gene and with a 6 bp barcode unique to each sample. The primers were (RP841F: 5′-GACTAGGGATTGGARTGG-3′, Reg1320R: 5′-AATTGCAAAGATCTATCCC-3′). After PCR amplification, all amplicon libraries were sequenced using an Illumina MiSeq PE 300 platform. We removed the barcodes and sequencing primers before data processing.

Eight samples (four per group) were selected to conduct shotgun metagenome sequencing. For each sample, 1 mg of genomic DNA was used with Illumina’s TruSeq for library preparation. Libraries were sequenced using an Illumina HiSeq PE 150 platform.

### 16S rRNA gene and 18S rRNA gene sequencing

We used FLASH (version 1.2.7) [43] to merge the paired-end reads generated from the DNA fragments. The data was processed using the QIIME (version 1.9.0) software package [44]. The sequences with a similarity level of more than 97% were clustered into OTUs using UPARSE (version 7.1; http://drive5.com/uparse/) [45]. Then, the representative sequences of these OTUs defined for the most abundant sequences were identified and respectively assigned to the bacteria and protozoa database of SILVA (version 11.9) [46].

### Shotgun metagenome sequencing

After sequencing, low-quality reads and contaminated adaptor and sheep host reads were removed from the raw sequenced reads by the FastQC software (version 0.11.8) [47] and BWA [48] package (version 0.7.12). Subsequently, the clean data reads were used as input for MEGAHIT (version 1.1.1) [49] with an option of --min-contig-len 500. After this, we removed the contig with a coverage of less than 60% using Salmon [50]. Prodigal (version 2.6.3) [51] was used to predict the contigs from each sample, and the ORFs derived from assembled contigs were maintained and clustered into a nonredundant data set by CD-HIT (version 4.6.7) [52] using a sequence identity cut-off of 0.95 [53]. Then, a pan-metagenome was constructed and used to analyse the changes in the metagenome function after starter feeding.

### Epithelial RNA extraction, sequencing, and qRT-PCR

The total RNA of the rumen epithelium across all samples was extracted using TRIzol (Takara Bio, Otsu, Japan) [54]. Then, a NanoDrop spectrophotometer (ND-1000UV-Vis; Thermo Fisher Scientific, Waltham, MA, USA) was used to quantify the RNA concentration, and the integrity of the RNA samples was evaluated using a 1.4% agarose-formaldehyde gel electrophoresis. After that, the concentration of each RNA sample was adjusted to 500 ng/μl per sample on account of optical density and then stored at − 80 °C. A total amount of 2 μg high-quality RNA per sample was used as input material for the RNA sample preparations. One migrogram of RNA was used for sequencing, sequencing libraries were generated using a NEBNext Ultra RNA Library Prep Kit for Illumina (E7530L, NEB, USA) following the manufacturer’s recommendations, and index codes were added to attribute sequences to each sample. Another 1 ug of RNA was reverse-transcribed using a PrimeScript® RT reagent Kit with a gDNA Eraser (Takara Bio, Shiga, Japan).

### Transcriptome sequencing and differentially expressed gene identification

We first used an inhouse perl script to remove low-quality reads. HISAT2 (https://ccb.jhu.edu/software/hisat2/index.shtml) [55] was used to align remaining reads to host. The software StringTie (version 1.3.4d) was used to map reads in order to estimate the expression of each gene transcript [56]. Gene expression levels were estimated by FPKM. Then, the differential gene expression between the CON and ST groups was estimated. Only genes with a Benjamini-Hochberg adjusted FDR < 0.05 and fold change > 2 were considered true DEGs (using DESeq2 [57]). The GO enrichment analysis of genes related to the DEGs was carried out by DAVID (version 6.8; https://david.ncifcrf.gov/) [58]. KOBAS (version 2.0) was used to test the statistical enrichment of DEGs in the KEGG pathways [59]. After selecting the eight genes that were enriched in the growth regulation modules involved in the growth-related signalling pathway, we conducted quantitative PCR to validate the expression and the primer sets used in our research, which are listed in Additional file 15: Table S13.

### Statistical analysis

All the significant differences in the present paper were tested using the Mann−Whiney *U* test in SPSS software (SPSS version 22.0, SPSS, Inc.). A value of *p* < 0.05 was regarded as statistically significant and *p* values were adjusted into FDR using the Benjamini-Hochberg method. The “Hmisc” package in the R software was used to complete the Spearman rank correlation coefficient. In addition, the “circlize” package was used to calculate the correlation between the dominant phylum or genus and carbohydrate-active enzymes classes. Cytoscape (version 3.5.1) [60] was performed for visualizing the gene-pathway-function co-regulation network. The parameters of some software and packages mentioned were showed in Additional file 16.

## Additional files

Additional file 1: **Table S1.** Compositions of the starter diet (DM basis). (DOCX 15 kb)

Additional file 2: **Table S2.** Effects of starter feeding on rumen fermentation in lambs. (DOCX 16 kb)

Additional file 3: **Table S3.** Effects of starter feeding on rumen epithelium papillae parameters in lambs. (DOCX 14 kb)

Additional file 4: **Figure S1.** The rarefaction of the rumen bacteria and ciliate protozoa based on the 16S rRNA and 18S rRNA genes in lambs. (PDF 295 kb)

Additional file 5: **Table S4.** The alpha diversity of rumen bacterial community based on 16S rRNA genes at 3% dissimilarity level. (DOCX 14 kb)

Additional file 6: **Table S5.** Effects of starter feeding on the relative abundance (%) of rumen bacteria at the phylum level. (DOCX 15 kb)

Additional file 7: **Table S6.** Effects of starter feeding on the relative abundance (%) of rumen bacteria at the genus level. (DOCX 17 kb)

Additional file 8: Effects of starter feeding on the relative abundance (%) of rumen ciliate protozoa at 3% dissimilarity level. (DOCX 15 kb)

Additional file 9: **Table S8.** Effects of starter feeding on the relative abundance (%) of rumen ciliate protozoa the at genus level. (DOCX 15 kb)

Additional file 10: **Table S9.** Effects of starter feeding on the relative abundance (TPM) of carbohydrate-active enzymes genes. (DOCX 15 kb)

Additional file 11: **Table S10.** Effects of starter feeding on the relative abundance (TPM) of GH family genes coded amylolytic enzymes. (DOCX 15 kb)

Additional file 12: **Figure S2.** Phylogenetic distribution of sequences of carbohydrate-active enzyme classes assigned to the top 10 phyla or genera. (PDF 1801 kb)

Additional file 13: **Table S11.** GO enrichment analysis of differentially expressed genes in biological processes. (XLSX 18 kb)

Additional file 14: **Table S12.** The expression profile (FPKM) of differentially expressed genes related to the cell growth module in the rumen epithelium of lambs. (DOCX 15 kb)

Additional file 15: **Table S13.** The primer sequences of differentially expressed genes related to the cell growth module in the rumen epithelium of lambs for qRT-PCR. (DOCX 16 kb)

Additional file 16. The parameters of the software and packages mentioned were presented. (DOCX 17 kb)

## Data Availability

Raw sequence reads for all samples described above were deposited into the NCBI Sequence Read Archive (SRA) database (project number, PRJNA512570 and accession number, SRP175061).

## Abbreviations

AA: Auxiliary activities
AK: Acetate kinase
ALDO: Fructose-bisphosphate aldolase
AMOVA: Analysis of molecular variance
BCD: Butyryl-CoA dehydrogenase
BHBD: 3-hydroxybutyryl-CoA dehydrogenase
BK: Butyrate kinase
CAZymes: Carbohydrate active enzymes
CB: Carbohydrate binding
CE: Carbohydrate esterases
CTFAB: Acetate-CoA transferase
DEGs: Differentially expressed genes
DHAP: Dihydroxyacetone phosphate
DMI: Dry matter intake
ECH: Enoyl-CoA hydratase
ENO: Enolase
FDR: False discovery rate
FPKM: Fragments per kilobase of transcript per million fragments mapped
G3P: Glyceraldehyde-3-phosphate
GAPDH: Glyceraldehyde 3-phosphate dehydrogenase
GH: Glycoside hydrolase
GO: Gene ontology
GPI: Glucose-6-phosphate isomerase
GT: Glycosyl transferase
HK: Hexokinase
KEGG: Kyoto Encyclopedia of Genes and Genomes
KO: KEGG Orthology
ORF: Open reading frame
OTUs: Operational taxonomic units
PBS: Phosphate-buffered saline
PCoA: Principal coordinate analysis
PEP: Phosphoenolpyruvate
PFK-1: 6-phosphofructokinase 1
PFOR: Pyruvate ferredoxin oxidoreductase
PGK: Phosphoglycerate kinase
PGM: Phosphoglycerate mutase
PK: Pyruvate kinase
PL: Polysaccharide lyases
PTA: Phosphate acetyltransferase
PTB: Phosphate butyryltransferase
THL: Acetyl-CoA C-acetyltransferase (thiolase)
TPI: Triosephosphate isomerase
TPM: Transcripts per million reads
VFA: Volatile fatty acid
