# Supplementary material for: Ruminal microbiome-host crosstalk stimulates the development of the ruminal epithelium in a lamb model
Source: Microbiome. 2019 Jun 3;7:83. doi: 10.1186/s40168-019-0701-y (PMC6547527; doi:10.1186/s40168-019-0701-y)
Supplement: Supplementary file 1 — Table S1. Compositions of the starter diet (DM basis). (DOCX 15 kb) [file 40168_2019_701_MOESM1_ESM.docx]

Table S1. Compositions of the starter diet (DM basis).

| Item | Starter feed |
| --- | --- |
| Ingredient composition (% DM) | |
| Cassava starch | 51.6 |
| Soybean meal | 28.0 |
| Corn gluten meal | 15.0 |
| Soybean oil | 1.2 |
| Limestone meal | 0.8 |
| CaHPO_4_ | 1.8 |
| NaCl | 0.6 |
| Premix^1^ | 1.0 |
| Nutrient composition |  |
| DM (%) | 88.37 |
| Metabolic energy^2^ (MJ/kg DM) | 11.31 |
| Crude protein^3^ (% DM) | 25.10 |
| Crude fat^3^ (% DM) | 3.55 |
| Crude fiber^3^ (% DM) | 6.24 |
| Crude ash^3^ (% DM) | 6.37 |
| Total starch (% DM) | 45.36 |

^1^Contained 16% calcium carbonate, 102 g/kg of Zn, 47 g/kg of Mn, 26 g/kg of Cu, 1,140 mg/kg of I, 500 mg/kg of Se, 340 mg/kg of Co, 17,167,380 IU/kg of vitamin A, 858,370 IU/kg of vitamin D, and 23,605 IU/kg of vitamin E;

^2^Values were calculated based on the database of NRC (2007);

^3^Values actually measure.
