# Supplementary material for: Ruminal microbiome-host crosstalk stimulates the development of the ruminal epithelium in a lamb model
Source: Microbiome. 2019 Jun 3;7:83. doi: 10.1186/s40168-019-0701-y (PMC6547527; doi:10.1186/s40168-019-0701-y)
Supplement: Supplementary file 2 — Table S2. Effects of starter feeding on rumen fermentation in lambs. (DOCX 16 kb) [file 40168_2019_701_MOESM2_ESM.docx]

Table S2. Effects of starter feeding on rumen fermentation in lambs.

| Ruminal parameters | CON | ST | *P* |
| --- | --- | --- | --- |
| Emptied rumen weight (g) | 184.7±12.77 | 220.5±11.67 | 0.034 |
| pH | 6.42±0.06 | 5.63±0.13 | <0.001 |
| Concentration (mM) |  |  |  |
| Total VFA | 115.54±3.62 | 150.59±11.48 | 0.034 |
| Acetate | 73.99±1.98 | 95.90±7.81 | 0.028 |
| Propionate | 25.77±1.12 | 29.36±3.23 | 0.650 |
| Butyrate | 10.08±0.76 | 18.95±2.45 | 0.007 |
| Others VFAs^1^ | 5.69±0.48 | 6.37±0. 82 | 0.496 |
| Proportion (%) |  |  |  |
| Acetate | 64.19±0.93 | 63.65±1.30 | 1.000 |
| Propionate | 22.27±0.61 | 19.59±1.70 | 0.151 |
| Butyrate | 8.65±0.48 | 12.51±1.26 | 0.019 |
| Other VFAs^1^ | 4.89±0.38 | 4.25±0.47 | 0.326 |
| Acetate: Propionate | 2.91±0.12 | 3.50±0.33 | 0.131 |

^1^Other VFAs, sum of valerate, isobutyrate and isovalerate. Values are means ± SEM, *n* = 10 per group.
