# Supplementary material for: Ruminal microbiome-host crosstalk stimulates the development of the ruminal epithelium in a lamb model
Source: Microbiome. 2019 Jun 3;7:83. doi: 10.1186/s40168-019-0701-y (PMC6547527; doi:10.1186/s40168-019-0701-y)
Supplement: Supplementary file 3 — Table S3. Effects of starter feeding on rumen epithelium papillae parameters in lambs. (DOCX 14 kb) [file 40168_2019_701_MOESM3_ESM.docx]

Table S3. Effects of starter feeding on rumen epithelium papillae parameters in lambs.

| Papillae morphology | CON | ST | *P* |
| --- | --- | --- | --- |
| Length (mm) | 1.57±0.08 | 2.56±0.15 | <0.001 |
| Width (mm) | 0.90±0.04 | 1.33±0.05 | <0.001 |
| Density (number/cm^2^) | 179±15 | 164±19 | 0.406 |
| Surface (mm^2^/cm^2^) | 512±56 | 1100±137 | 0.002 |

Values are means ± SEM, *n* = 10 per group.
