# Supplementary figures and images for: Ruminal microbiome-host crosstalk stimulates the development of the ruminal epithelium in a lamb model
Source: Microbiome. 2019 Jun 3;7:83. doi: 10.1186/s40168-019-0701-y (PMC6547527; doi:10.1186/s40168-019-0701-y)

**A**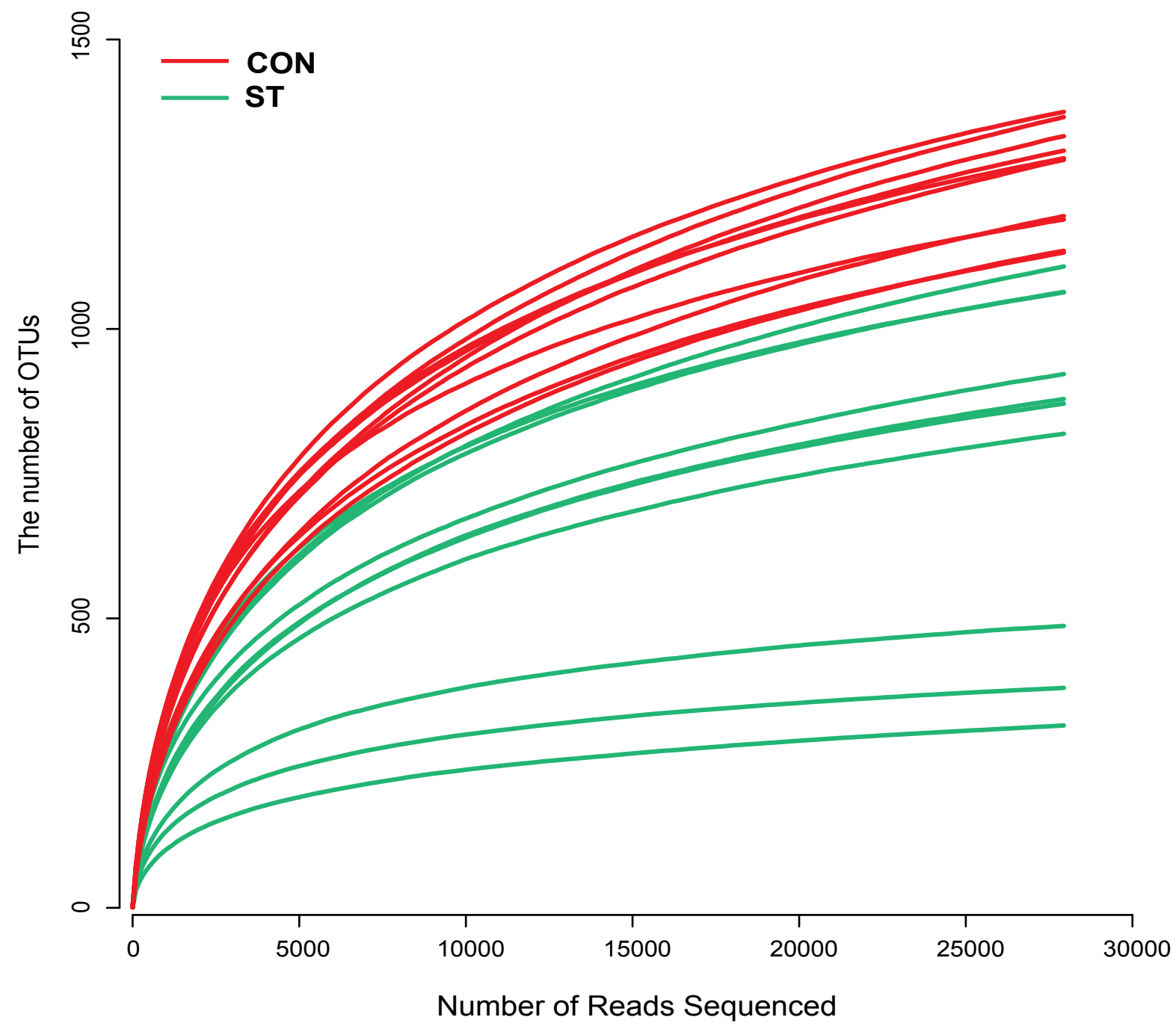**B**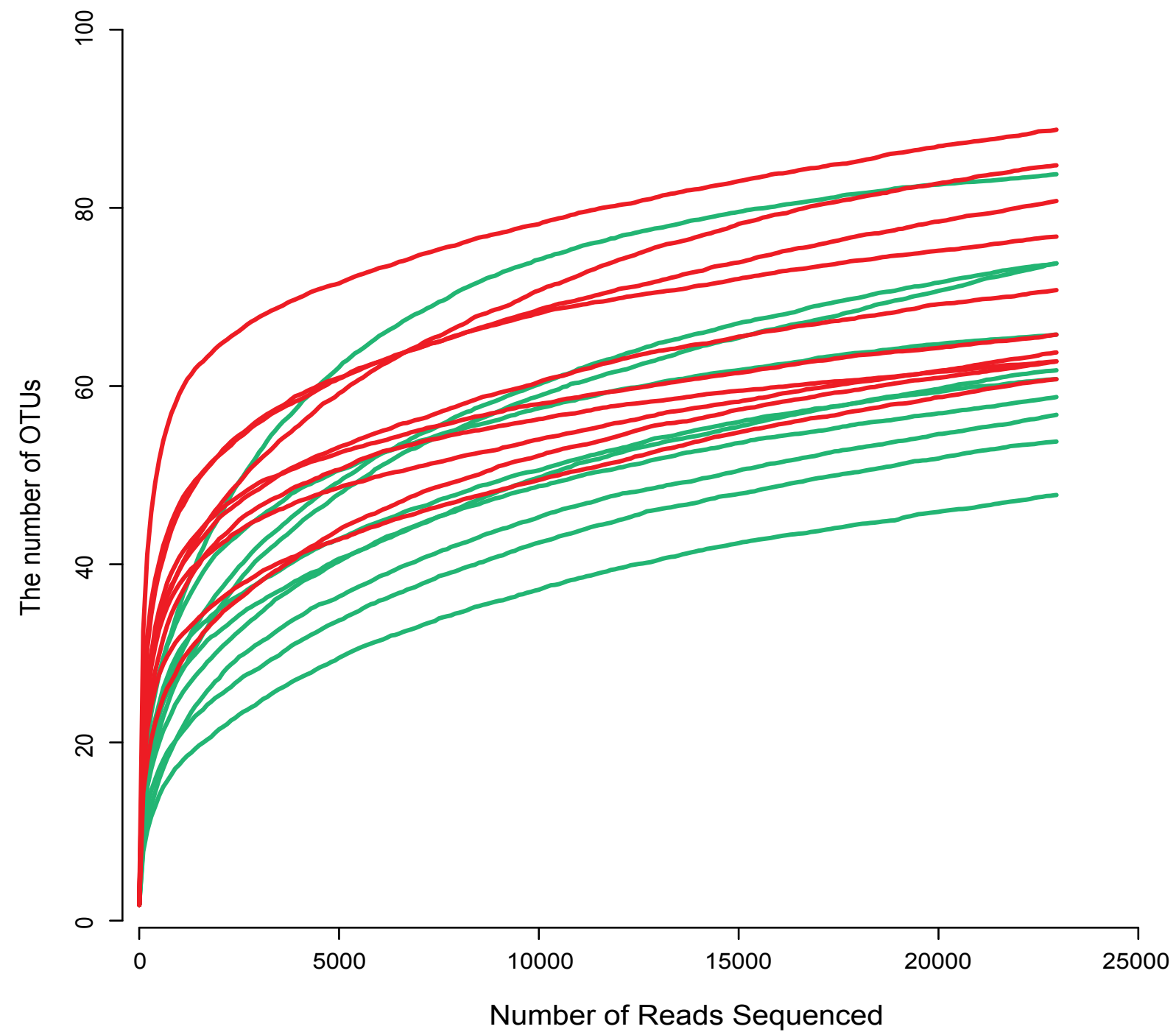

Supplement: Supplementary file 4 — Figure S1. The rarefaction of the rumen bacteria and ciliate protozoa based on the 16S rRNA and 18S rRNA genes in lambs. (PDF 295 kb) [file 40168_2019_701_MOESM4_ESM.pdf]

A

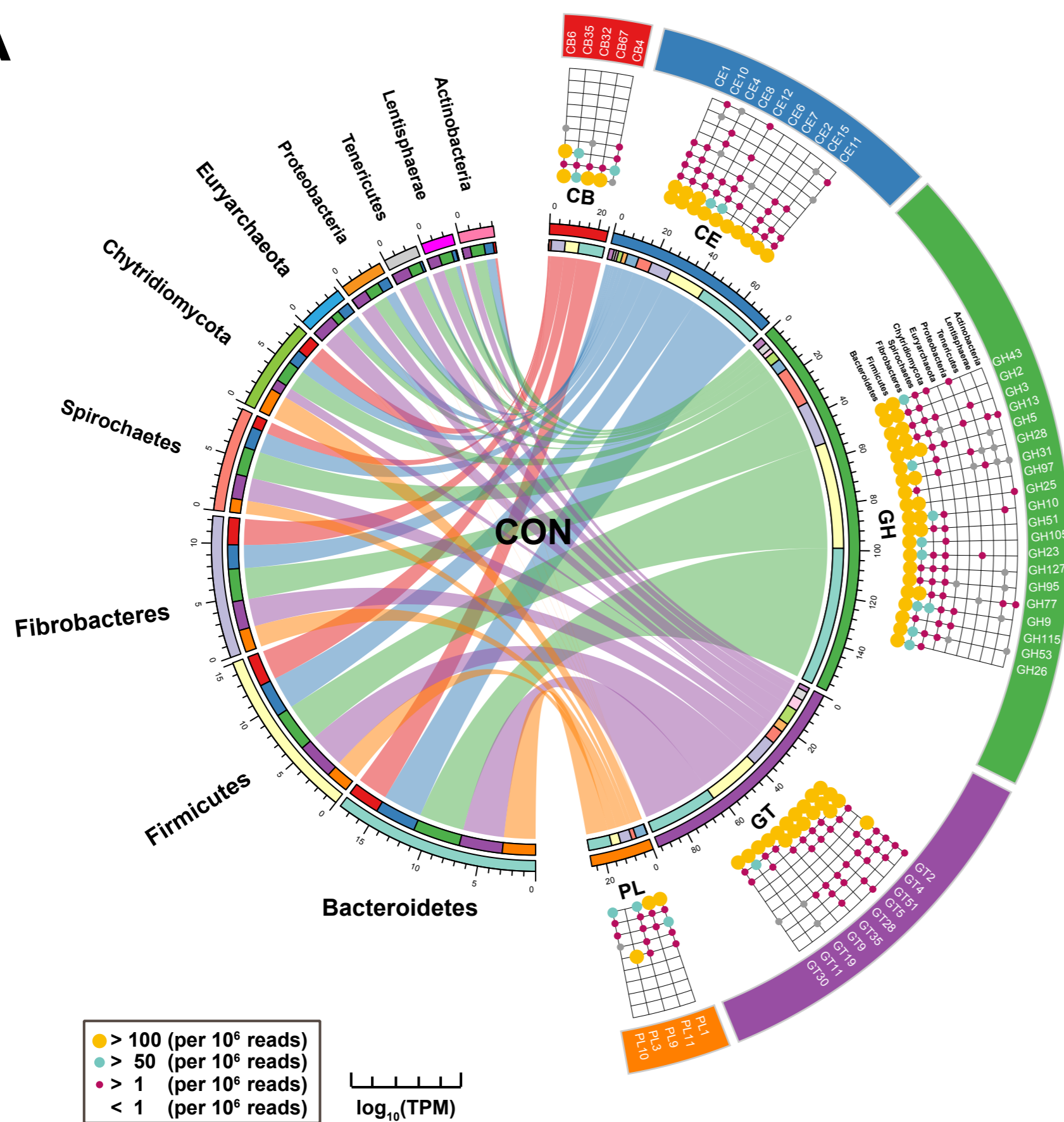

Phylum

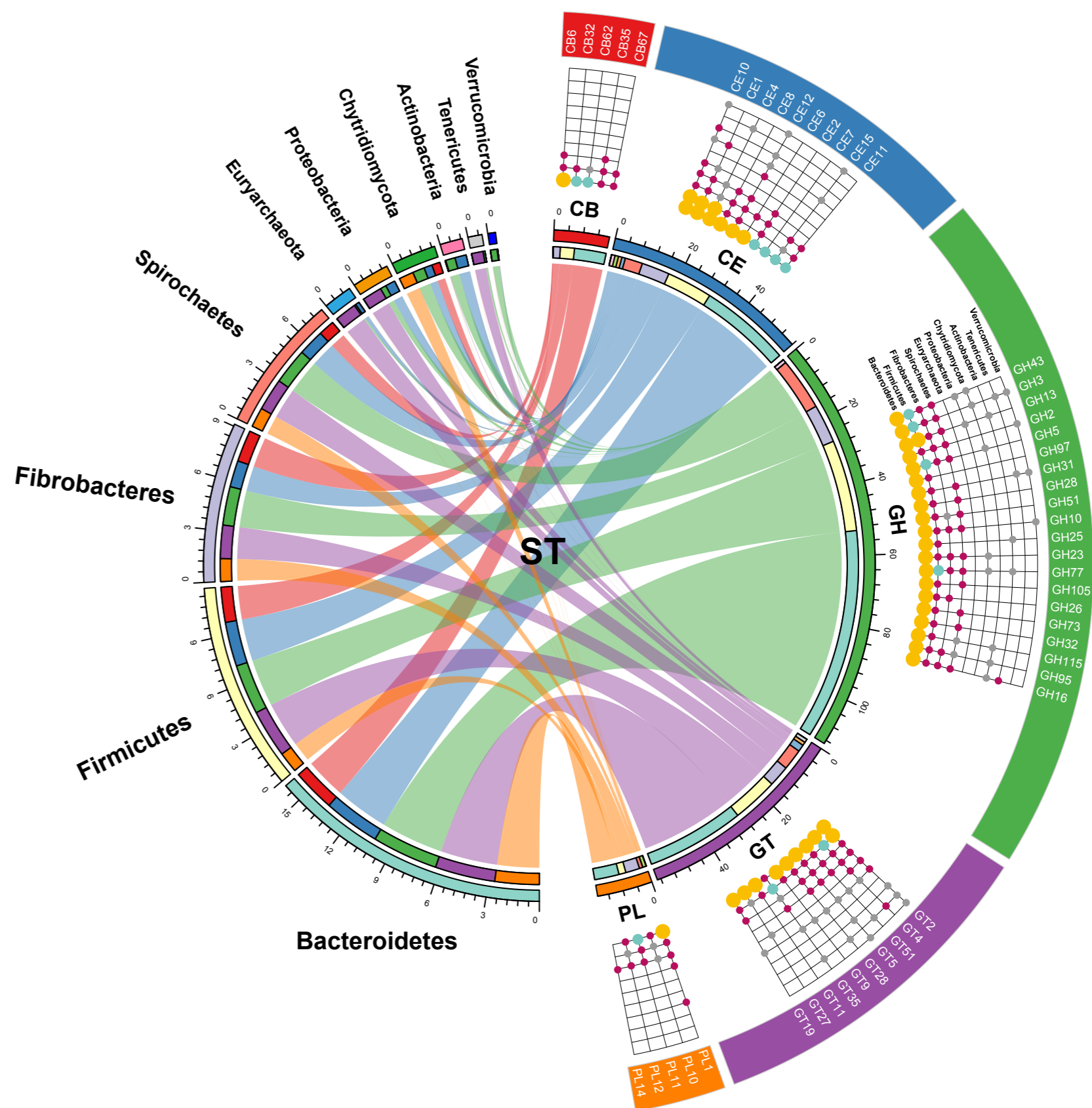

B

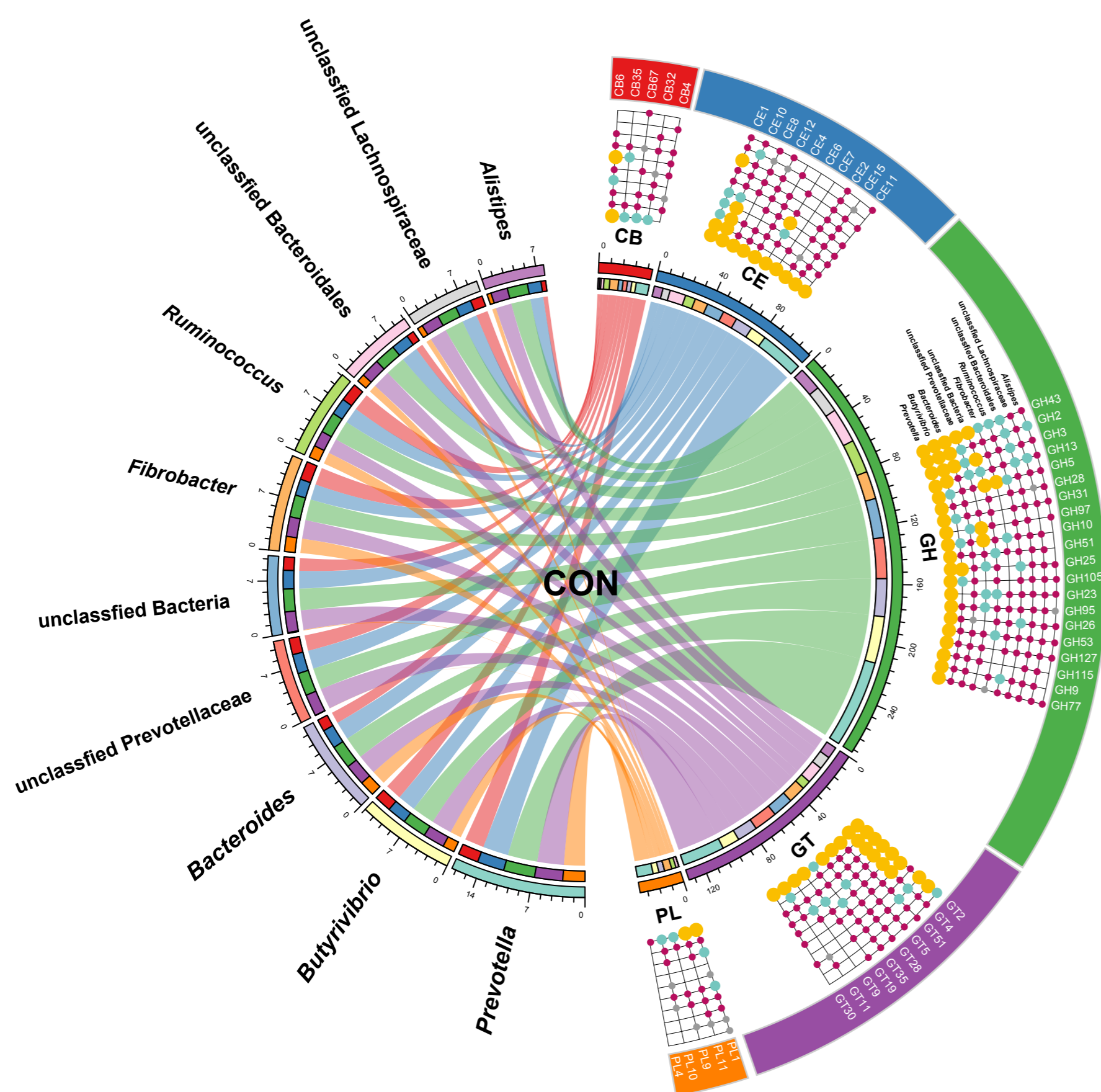

Genus

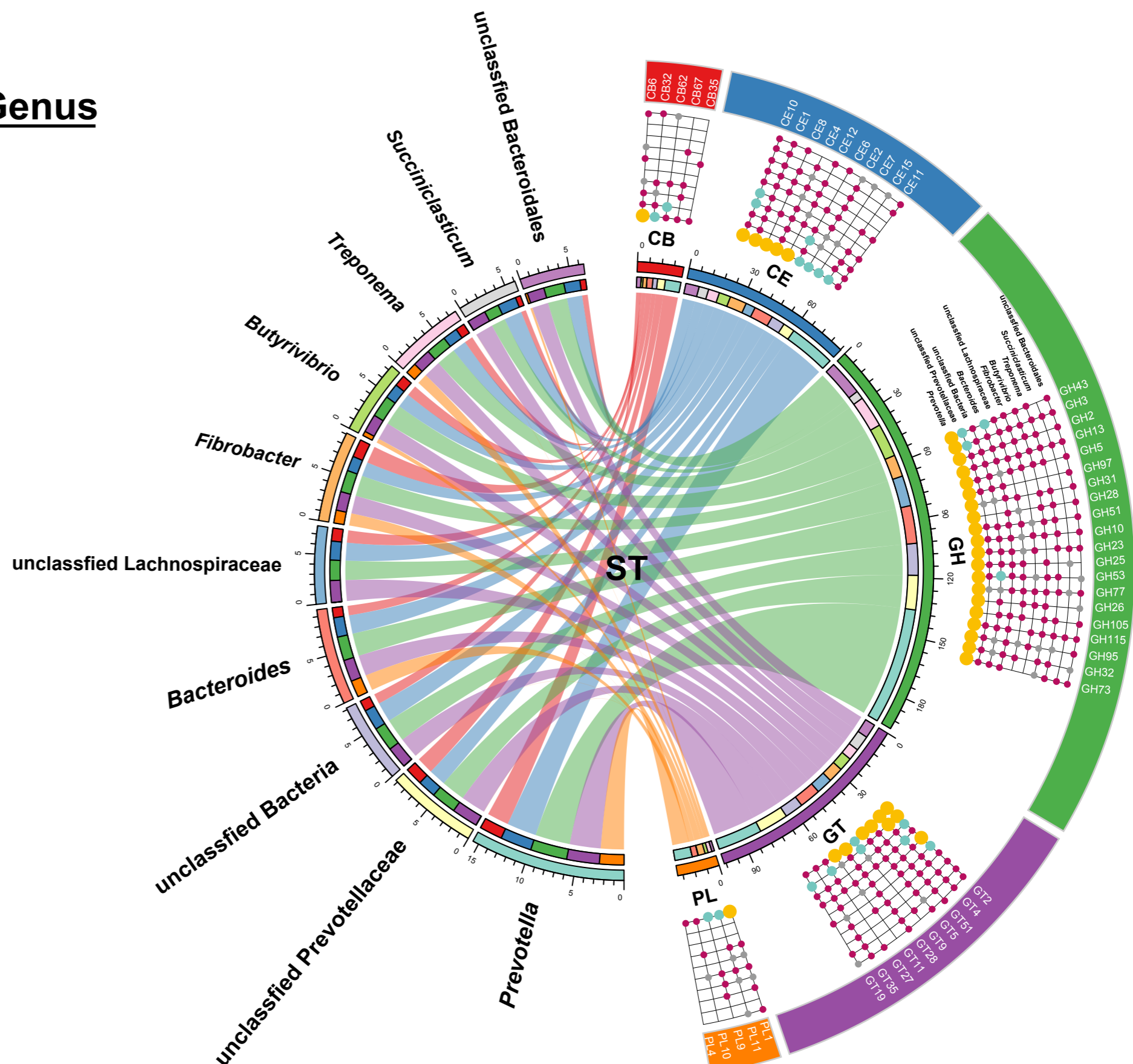

Supplement: Supplementary file 12 — Figure S2. Phylogenetic distribution of sequences of carbohydrate-active enzyme classes assigned to the top 10 phyla or genera. (PDF 1801 kb) [file 40168_2019_701_MOESM12_ESM.pdf]
