# Supplementary material for: Ruminal microbiome-host crosstalk stimulates the development of the ruminal epithelium in a lamb model
Source: Microbiome. 2019 Jun 3;7:83. doi: 10.1186/s40168-019-0701-y (PMC6547527; doi:10.1186/s40168-019-0701-y)
Supplement: Supplementary file 5 — Table S4. The alpha diversity of rumen bacterial community based on 16S rRNA genes at 3% dissimilarity level. (DOCX 14 kb) [file 40168_2019_701_MOESM5_ESM.docx]

Table S4. The alpha diversity of rumen bacterial community based on 16S rRNA gene at 3% dissimilarity level.

| Alpha diversity | CON | ST | *P* |
| --- | --- | --- | --- |
| Observed OTUs | 1262±29 | 791±92 | <0.001 |
| Chao 1 | 1560±38 | 1002±121 | <0.001 |
| ACE | 1557±37 | 974±117 | <0.001 |
| Shannon | 5.33±0.093 | 4.44±0.202 | 0.001 |
| Simpson | 0.02±0.003 | 0.05±0.009 | 0.019 |

*Standardizing sequences depth at 27,942. Values are means ± SEM, *n* = 10 per group.
