# Supplementary material for: Ruminal microbiome-host crosstalk stimulates the development of the ruminal epithelium in a lamb model
Source: Microbiome. 2019 Jun 3;7:83. doi: 10.1186/s40168-019-0701-y (PMC6547527; doi:10.1186/s40168-019-0701-y)
Supplement: Supplementary file 6 — Table S5. Effects of starter feeding on the relative abundance (%) of rumen bacteria at the phylum level. (DOCX 15 kb) [file 40168_2019_701_MOESM6_ESM.docx]

Table S5. Effects of starter feeding on the relative abundance (%) of rumen bacteria at phylum level.

| Phylum | CON | ST | SEM | *P* |
| --- | --- | --- | --- | --- |
| Bacteroidetes | 62.35 | 60.15 | 1.840 | 0.545 |
| Firmicutes | 34.05 | 33.50 | 1.868 | 0.940 |
| Spirochaetae | 1.15 | 2.28 | 0.333 | 0.257 |
| Proteobacteria | 0.63 | 1.21 | 0.145 | 0.034 |
| Tenericutes | 0.52 | 0.29 | 0.064 | 0.021 |
| Actinobacteria | 0.05 | 1.32 | 0.280 | 0.002 |

Only the dominant phyla with a mean relative abundance more than 0.5% in one group were listed.
