# Supplementary material for: Ruminal microbiome-host crosstalk stimulates the development of the ruminal epithelium in a lamb model
Source: Microbiome. 2019 Jun 3;7:83. doi: 10.1186/s40168-019-0701-y (PMC6547527; doi:10.1186/s40168-019-0701-y)
Supplement: Supplementary file 7 — Table S6. Effects of starter feeding on the relative abundance (%) of rumen bacteria at the genus level. (DOCX 17 kb) [file 40168_2019_701_MOESM7_ESM.docx]

Table S6. Effects of starter feeding on the relative abundance (%) of rumen bacteria at genus level.

| Genus | CON | ST | SEM | *P* |
| --- | --- | --- | --- | --- |
| *Prevotella* | 34.75 | 35.27 | 2.820 | 0.880 |
| *RC9_gut_group* | 9.09 | 4.16 | 0.945 | 0.007 |
| Unclassfied Christensenellaceae | 7.74 | 2.28 | 0.881 | 0.001 |
| Unclassfied Prevotellaceae | 7.52 | 5.25 | 0.846 | 0.406 |
| Unclassfied Ruminococcaceae | 6.38 | 6.06 | 0.658 | 0.940 |
| Unclassfied S24-7 | 6.02 | 8.27 | 1.618 | 0.821 |
| Unclassfied Lachnospiraceae | 4.16 | 0.87 | 0.521 | <0.001 |
| Unclassfied BS11_gut_group | 3.97 | 5.13 | 0.659 | 0.705 |
| *Succiniclasticum* | 3.43 | 4.25 | 0.618 | 0.940 |
| *Butyrivibrio* | 3.01 | 1.85 | 0.318 | 0.049 |
| *Treponema* | 1.11 | 2.24 | 0.329 | 0.226 |
| *Ruminococcus* | 1.09 | 4.29 | 0.673 | 0.082 |
| *Megasphaera* | <0.01 | 2.56 | 0.803 | 0.017 |
| *Sharpea* | <0.01 | 1.55 | 0.391 | <0.001 |
| *Dialister* | <0.01 | 1.40 | 0.429 | 0.013 |
| Unclassified Veillonellaceae | 0.69 | 1.11 | 0.148 | 0.241 |
| *Roseburia* | 0.42 | 1.05 | 0.166 | 0.130 |
| *Selenomonas* | 0.37 | 0.97 | 0.227 | 0.199 |
| Unclassified Bifidobacteriaceae | <0.01 | 0.96 | 0.273 | 0.005 |
| *Saccharofermentans* | 0.72 | 0.72 | 0.130 | 0.450 |
| *Blautia* | 0.71 | 0.39 | 0.086 | 0.070 |
| *Alloprevotella* | 0.18 | 0.70 | 0.124 | 0.064 |
| *Mitsuokella* | 0.01 | 0.62 | 0.156 | 0.009 |
| *Oribacterium* | 0.60 | 0.16 | 0.131 | 0.007 |
| *Quinella* | 0.59 | <0.01 | 0.116 | <0.001 |

Only the dominant genera with a mean relative abundance more than 0.5% in one group were listed.
