# Supplementary material for: Ruminal microbiome-host crosstalk stimulates the development of the ruminal epithelium in a lamb model
Source: Microbiome. 2019 Jun 3;7:83. doi: 10.1186/s40168-019-0701-y (PMC6547527; doi:10.1186/s40168-019-0701-y)
Supplement: Supplementary file 8 — Effects of starter feeding on the relative abundance (%) of rumen ciliate protozoa at 3% dissimilarity level. (DOCX 15 kb) [file 40168_2019_701_MOESM8_ESM.docx]

Table S7. The alpha diversity of rumen ciliate protozoa based on 18S rRNA genes at 3% dissimilarity level.

| Alpha diversity | CON | ST | *P* |
| --- | --- | --- | --- |
| Observed OTUs | 71±3 | 63±3 | 0.069 |
| Chao 1 | 82±3 | 76±5 | 0.226 |
| ACE | 94±6 | 75±4 | 0.021 |
| Shannon | 2.41±0.133 | 1.49±0.183 | 0.001 |
| Simpson | 0.19±0.276 | 0.42±0. 676 | 0.007 |

*Standardizing sequences depth at 22952. Values are means ± SEM, *n* = 10 per group.
