# Supplementary material for: Ruminal microbiome-host crosstalk stimulates the development of the ruminal epithelium in a lamb model
Source: Microbiome. 2019 Jun 3;7:83. doi: 10.1186/s40168-019-0701-y (PMC6547527; doi:10.1186/s40168-019-0701-y)
Supplement: Supplementary file 9 — Table S8. Effects of starter feeding on the relative abundance (%) of rumen ciliate protozoa the at genus level. (DOCX 15 kb) [file 40168_2019_701_MOESM9_ESM.docx]

Table S8. Effects of starter feeding on the relative abundance (%) of rumen ciliate protozoa at genus level.

| Genus | CON | ST | SEM | *P* | |  |
| --- | --- | --- | --- | --- | --- | --- |
| *Diplodinium* | 1.12 | 0.08 | 0.242 | | 0.023 | |
| *Entodinium* | 54.05 | 83.00 | 6.508 | | 0.010 | |
| *Isotricha* | 0.79 | 0.06 | 0.385 | | 0.522 | |
| *Ophryoscolex* | 5.77 | 0.65 | 1.380 | | 0.023 | |
| *Polyplastron* | 20.65 | 12.28 | 4.547 | | 0.034 | |
| Unclassified Trichostomatia | 17.27 | 3.92 | 3.261 | | 0.002 | |

Only the dominant genera with a mean relative abundance more than 0.5% in one group were listed.
