# Supplementary material for: Ruminal microbiome-host crosstalk stimulates the development of the ruminal epithelium in a lamb model
Source: Microbiome. 2019 Jun 3;7:83. doi: 10.1186/s40168-019-0701-y (PMC6547527; doi:10.1186/s40168-019-0701-y)
Supplement: Supplementary file 10 — Table S9. Effects of starter feeding on the relative abundance (TPM) of carbohydrate-active enzymes genes. (DOCX 15 kb) [file 40168_2019_701_MOESM10_ESM.docx]

Table S9. Effects of starter feeding on the relative abundance (TPM) of carbohydrate-active enzymes genes.

| CAZy | CON | ST | *P* |
| --- | --- | --- | --- |
| Total CAZy | 23856.33±994.97 | 20780.84±255.32 | 0.021 |
| AA | 20.67±1.72 | 26.98±3.04 | 0.083 |
| CB | 901.50±73.99 | 740.18±53.52 | 0.149 |
| CE | 3310.68±179.10 | 2727.32±102.04 | 0.043 |
| GH | 12095.42±634.39 | 10435.01±164.66 | 0.021 |
| GT | 6839.15±177.23 | 6438.18±18.19 | 0.021 |
| PL | 684.69±51.12 | 407.72±28.00 | 0.021 |

AA auxiliary activities, CB carbohydrate binding, CE carbohydrate esterases, GH glycoside hydrolase, GT glycosyl transferase, PL polysaccharide lyases. Values are means ± SEM, *n* = 4 per group.
