# Supplementary material for: Ruminal microbiome-host crosstalk stimulates the development of the ruminal epithelium in a lamb model
Source: Microbiome. 2019 Jun 3;7:83. doi: 10.1186/s40168-019-0701-y (PMC6547527; doi:10.1186/s40168-019-0701-y)
Supplement: Supplementary file 11 — Table S10. Effects of starter feeding on the relative abundance (TPM) of GH family genes coded amylolytic enzymes. (DOCX 15 kb) [file 40168_2019_701_MOESM11_ESM.docx]

Table S10. Effects of starter feeding on the relative abundance (TPM) of GH family genes coded amylolytic enzymes.

| Amylolytic enzymes | CAZy class | CON | ST | *P* |
| --- | --- | --- | --- | --- |
| α-amylase | GH13 | 840.79±30.30 | 1081.19±74.65 | 0.021 |
|  | GH31 | 378.36±23.44 | 329.38±9.20 | 0.149 |
|  | GH57 | 95.13±5.39 | 89.92±4.01 | 0.546 |
|  | G77 | 204.16±13.82 | 211.55±4.50 | 1.000 |
|  | GH119 | 0.12± 0.07 | 1.71±1.20 | 0.248 |
| β-amylase | GH14 | 0 | 0.11±0.11 | 0.317 |
| Glucoamylase | GH15 | 4.27±0.27 | 3.81±0.94 | 0.248 |

Values are means ± SEM, *n* = 4 per group.
