# Supplementary material for: Ruminal microbiome-host crosstalk stimulates the development of the ruminal epithelium in a lamb model
Source: Microbiome. 2019 Jun 3;7:83. doi: 10.1186/s40168-019-0701-y (PMC6547527; doi:10.1186/s40168-019-0701-y)
Supplement: Supplementary file 14 — Table S12. The expression profile (FPKM) of differentially expressed genes related to the cell growth module in the rumen epithelium of lambs. (DOCX 15 kb) [file 40168_2019_701_MOESM14_ESM.docx]

Table S12. The expression profile (FPKM) of differentially expressed genes related to the cell growth module in the rumen epithelium of lambs.

| Gene | CON | ST | FDR | Log_2_FC |
| --- | --- | --- | --- | --- |
| MAPK1 | 6.19±2.48 | 24.16±2.34 | 0.008 | 1.64 |
| PIK3CB | 1.83±0.70 | 9.95±1.25 | <0.001 | 2.20 |
| SAV1 | 1.68±0.87 | 7.99±0.87 | 0.004 | 2.01 |
| SNAI2 | 14.41±4.18 | 40.63±1.56 | 0.048 | 1.20 |
| DLG1 | 2.58±1.42 | 11.40±1.00 | 0.045 | 1.45 |
| ITGA6 | 7.98±2.44 | 21.14±2.14 | 0.040 | 1.16 |
| TNFSF10 | 0.65±0.20 | 3.73±0.79 | 0.009 | 2.16 |
| BAD | 67.52±5.50 | 38.97±2.66 | 0.022 | -1.16 |

Values are means ± SEM, *n* = 10 per group.
