# Supplementary material for: Ruminal microbiome-host crosstalk stimulates the development of the ruminal epithelium in a lamb model
Source: Microbiome. 2019 Jun 3;7:83. doi: 10.1186/s40168-019-0701-y (PMC6547527; doi:10.1186/s40168-019-0701-y)
Supplement: Supplementary file 15 — Table S13. The primer sequences of differentially expressed genes related to the cell growth module in the rumen epithelium of lambs for qRT-PCR. (DOCX 16 kb) [file 40168_2019_701_MOESM15_ESM.docx]

Table S13. The primer sequences of differentially expressed genes related to the cell growth module in the rumen epithelium of lambs for qRT-PCR.

| Gene Name | Gene ID | Primer sequence (5’→3’) | Amplicon Size (bp) |
| --- | --- | --- | --- |
| MAPK1 | XM_015103608.1 | For: TACTGCCAGAGGACGCTGAGAG  R: AGATGTGGTCGTTGCTGAGGTG | 190 |
| PIK3CB | XM_004003302.3 | For: TGGCTGCTTAGCAACAGGAGAC  R: TCAATGGCTCGGTCCAGGTCAT | 180 |
| SAV1 | XM_004010517.3 | For: ACCAGCCACAGCAAACCGAAAG  R: TGTGTCCAGGTCAGCCAGTTGA | 161 |
| SNAI2 | NM_001126342.1 | For: TTCGGACGCACACCTTACCTTG  R: AGCCCTCAGATTGGACCTGTCT | 158 |
| DLG1 | XM_012096585.1 | For: ACACCAGCATCACCAGCCAGAT  R: TAAGATCGGCAGGTCCTCCAGC | 190 |
| ITGA6 | XM_015093358.1 | For: GCAGCGTGAACGTGAACTGTGT  R: GGTGGCGGCAGTGACATCAATG | 173 |
| TNFSF10 | XM_012099171.2 | For: GACAGTTCTGCGTGCTGATCCT  R: CACCACTTGTCACCAGAATGTT | 138 |
| BAD | XM_004019650.3 | For: TTTCGGAAGACTGAGGTCTGAT  R: CGGCGAAGTTAGGGTTAATCTC | 185 |
| GAPDH | NM_001190390.1 | For: GGGTCATCATCTCTGCACCT  R: GGTCATAAGTCCCTCCACGA | 180 |
