# Supplementary material for: Ruminal microbiome-host crosstalk stimulates the development of the ruminal epithelium in a lamb model
Source: Microbiome. 2019 Jun 3;7:83. doi: 10.1186/s40168-019-0701-y (PMC6547527; doi:10.1186/s40168-019-0701-y)
Supplement: Supplementary file 16 — The parameters of the software and packages mentioned were presented. (DOCX 17 kb) [file 40168_2019_701_MOESM16_ESM.docx]

**The parameters of some software and packages mentioned were presented.**

**Software:** FastQC (version 0.11.8)

**Parameters:**

java -jar /mnt/sdb/bin/Trimmomatic-0.33//trimmomatic-0.33.jar PE -threads 40 -phred33 input.samplename.R1.fastq.gz input.samplename.R2.fastq.gz output.samplename.clip.1.fq.gz output.samplename.single.R1.fastq.gz output.samplename.clip.2.fq.gz output.samplename.single.R2.fastq.gz ILLUMINACLIP:/mnt/sdb/bin/Trimmomatic-0.33//adapters/TruSeq2-PE.fa:2:30:10 SLIDINGWINDOW:4:15 MINLEN:75

**Software:** MEGAHIT (version 1.1.1)

**Parameters:**

megahit -t 40 -1 input.samplename.clean.1.fq.gz -2 input.samplename.clean.2.fq.gz --min-contig-len 500 -o output.samplename-megahit

**Software:** Prodigal (version 2.6.3)

**Parameters:**

prodigal -a input.samplename.faa -i input.samplename.contigs -f gff -o output.samplename.gff -p meta -q -d input.samplename.ffn

**Software:** CD-HIT (version 4.6.7)

**Parameters:**

cd-hit-est -i All_samples.ffn -o All_samples.geneSet.ffn -n 9 -c 0.95 -G 0 -M 0 -d 0 -aS 0.9 -r 1 -T 140

**Software:** StringTie (version 1.3.4d)

**Parameters:**

stringtie input.bam -G guide_gff -l STRG -o out_gtf -p 12 -m 200 -j 1 -f 0.1 -c 2.5 -g 50

**Package:** DESeq2

**Parameters:** DESeq (object, test = "Wald", fitType = "local")

**Description:**

Using the DESeqDataSetFromMatrix function, the gene expression matrix (calculating by the htseq software and filtering the genes using the CPM indicator) and the sample grouping table were as the input file to obtain the object variable, and then the object variable was used for DESeq function to perform the difference significance test. The core parameter “test” set as “wald”, which means that *P*-value was calculated using nbinomWaldTest (negative binomial test). In addition, *P*-value was corrected using the FDR algorithm.

**Software:** KOBAS 2.0

**Parameters:**

kobas-2.0/scripts/identify.py -f input.DEG.ko -n BH -b sheep.pathway.txt -o output.ko.enrich.xls
